# Integrated Transcriptomic and Metabolomic Profiling Identifies Candidate Genes and Pathways Associated With Pedicel Abscission Susceptibility in *Capsicum annuum*

**DOI:** 10.1002/fsn3.70571

**Published:** 2025-07-24

**Authors:** Lei He, Xi Yan, Di Wu, Sha Yang, Wei Lai, Chongzheng Liu, Hong Yang, Jianwen He

**Affiliations:** ^1^ Pepper Research Institute, Guizhou Academy of Agricultural Sciences Guiyang China; ^2^ Guizhou Key Laboratory of Molecular Breeding for Characteristic Horticultural Crops Guiyang China; ^3^ College of Horticulture, Hunan Agricultural University Changsha China

**Keywords:** *Capsicum annuum*, metabolomic, pedicel abscission, susceptibility, transcriptomic

## Abstract

Pedicel abscission susceptibility in *Capsicum annuum* affects fruit retention and harvesting efficiency, making it a key agronomic trait in pepper breeding. In this study, two germplasm lines (LC‐8 and LC‐17) exhibiting distinct abscission characteristics were analyzed to explore the molecular basis underlying this trait. Although phenotypic differences have been documented, the molecular mechanisms regulating abscission susceptibility remain largely unclear. We performed a comprehensive analysis integrating morphological observations with transcriptomic and metabolomic profiling. Anatomical analysis revealed differential lignification patterns in the abscission zone (AZ), suggesting structural specialization. Transcriptome profiling identified 4635–7519 differentially expressed genes (DEGs), with KEGG enrichment highlighting phenylpropanoid biosynthesis and plant hormone signal transduction as core regulatory pathways. Metabolomic profiling detected 966 metabolites, including significantly altered flavonoids (e.g., apigenin O‐hexosyl‐O‐pentoside, naringenin O‐malonylhexoside) and phytohormones (e.g., abscisic acid, GA_7‐1_). Integrated multi‐omics analysis revealed that key genes and metabolites involved in lignin biosynthesis and hormone signaling displayed distinct expression and accumulation patterns between the two lines. These findings suggest that these metabolic pathways play central roles in modulating pedicel abscission susceptibility. This study lays a theoretical foundation for regulating abscission and supports the breeding of pepper cultivars optimized for mechanical harvesting.

## Graphical abstract

*Capsicum* germplasm lines with distinct pedicel abscission behaviors were analyzed via integrated transcriptomics and metabolomics. Differential lignification patterns, hormone signaling, and flavonoid biosynthesis were linked to abscission susceptibility. Key regulators of lignin and phytohormone pathways underpin structural and metabolic control of the abscission zone.

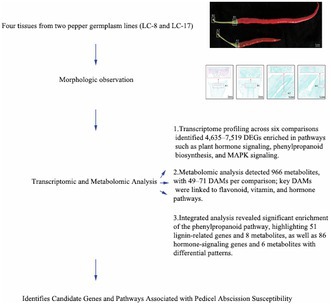

## 1 Introduction

Pepper ( *Capsicum annuum* L.), a member of the Solanaceae family, is considered one of the most important vegetables globally (Carrizo García et al. 2016; Feng et al. 2020). It is abundant in vitamins, capsaicin, capsidiol, minerals, and protein (Olatunji and Afolayan 2018). In China, peppers are consumed and processed through a variety of methods (Ye et al. 2020; Song et al. 2022; Chen et al. 2024). However, the cultivation and processing of peppers are characterized by significant labor intensity and time consumption, frequently necessitating manual harvesting and the skilled detachment of fruit pedicels. The asynchronous ripening of peppers precludes the feasibility of relying solely on mechanized, single‐instance harvesting, thereby requiring multiple iterations of manual selection to ensure the collection of mature fruits (Arad et al. 2020; Safri et al. 2024). Consequently, the development of pepper varieties that facilitate easy detachment from their pedicels is of particular importance.

In the plant kingdom, organ abscission is a prevalent physiological phenomenon characterized by the detachment of plant organs such as leaves, flowers, and fruits from their parent structure (Sundaresan et al. 2015; Patharkar and Walker 2018; Li and Su 2024). This process is delineated into four distinct phases: the formation of the abscission zone (AZ), the response to abscission signals, the activation of abscission, and the formation of a protective layer following abscission (Sundaresan et al. 2015; Patterson 2001; Patterson et al. 2016). Although abscission results in the separation of an entire organ from the plant, the underlying processes are orchestrated by cells within a localized zone (Roberts et al. 2000). Within this zone, the mechanical weakening of cell walls is facilitated by enzymes that degrade the middle lamella, including cellulases, polygalacturonases, and pectin methyl esterases, among others (Bleecker and Patterson 1997; Roberts et al. 2002). This process represents a highly coordinated event involving various mechanisms such as cellular structural changes, hormone regulation, and gene expression (Estornell et al. 2013; Nakano et al. 2013).

Significant advancements have been achieved in the study of plant hormones, particularly regarding their roles in abscission. Ethylene is a master regulator of abscission, as demonstrated by its activation of cell wall degradation genes in lychee fruit stalks (Ma et al. 2020). Furthermore, ethylene interacts with jasmonate and abscisic acid, thereby influencing both fruit maturation and abscission (Alferez et al. 2021). The polar transport of auxin is identified as a crucial regulatory mechanism; in grapevines, it has been shown to reduce the expression of ethylene‐related genes and mitigate fruit drop (Kühn et al. 2014). Additionally, auxin interacts with abscisic acid in the context of abscission (Shalom et al. 2014). Abscisic acid itself plays a significant role in fruit pedicel abscission by regulating the expression of cell wall degradation proteins. In sweet cherries, the accumulation of abscisic acid is correlated with abscission, and the ratio of abscisic acid to auxin emerges as a critical regulatory factor (Qiu et al. 2021; Cai et al. 2018). In the domain of genetic research, significant progress has been made in identifying genes associated with abscission in Arabidopsis, including *BLADE ON PETIOLE1*/*2* (*BOP1*/*2*) (McKim et al. 2008), *KNAT/BP* (Wang et al. 2006), *INFLORESCENCE DEFICIENT IN ABSCISSION* (IDA) (Butenko et al. 2003), *DAB* (Patterson and Bleecker 2004), *HAESA/HSL2* (Gubert and Liljegren 2014), *AtZFP2* (Cai and Lashbrook 2008), and *ARP4*/*ARP7* (Kandasamy et al. 2005). Mao et al. have explored the role of *JOINTLESS*, a MADS‐box gene, in regulating the development of the abscission zone in tomato flowers (Mao et al. 2000). Furthermore, Kai et al. have proposed that *SlPYL9* influences flower abscission and fruit ripening in tomatoes via ABA signaling pathways (Kai et al. 2019). Yan et al. have identified a novel function of *SlBL4*, which is essential for fruit pedicel organogenesis and abscission in tomatoes (Yan et al. 2021). Additionally, Gao et al. have indicated that RhIAA16 is a key factor in the abscission of rose petals (Gao et al. 2016). Although preliminary studies have identified certain genes associated with abscission and have explored their underlying mechanisms, targeted investigations specifically addressing abscission susceptibility have not yet been conducted.

Transcriptomics enables systematic mapping of RNA dynamics, distinguishing protein‐coding mRNAs from regulatory non‐coding RNAs to identify pathway‐level perturbations (Guttman and Rinn 2012). In conjunction with this method, metabolomics provides a comprehensive characterization of the chemically diverse small‐molecule metabolites (less than 1500 Da) present in biological systems (Patti et al. 2012; Rinschen et al. 2019). Liu et al. elucidated the mechanisms of color formation through a correlative analysis of flavonoid and carotenoid metabolites alongside their biosynthetic gene expression profiles (Liu et al. 2020). Zhang et al. revealed cold stress adaptation mechanisms through integrated profiling of CBF transcriptional regulators and osmoprotective metabolites (e.g., polyamines) (Zhang et al. 2022). Wang et al. defined anthracnose resistance as a synergistic activation of phenylpropanoid biosynthesis genes and jasmonate signaling hubs via comparative transcriptomics (Wang et al. 2024).

In this study, we conducted an extensive transcriptomic and metabolomic analysis on two Capsicum germplasm lines: LC‐8 demonstrates two opposing characteristics: a high susceptibility to pedicel abscission, yet a decreased susceptibility to abscission at the pedicel‐stem junction. Conversely, LC‐17 exhibits a reduced susceptibility to pedicel abscission while displaying an increased susceptibility to abscission at the pedicel‐stem junction under the same conditions. The objective was to identify candidate pathways and genes that influence abscission susceptibility. This research offers novel targets and theoretical foundations for molecular breeding.

## 2 Materials and Methods

### 2.1 Plant Materials

**FIGURE 1:**
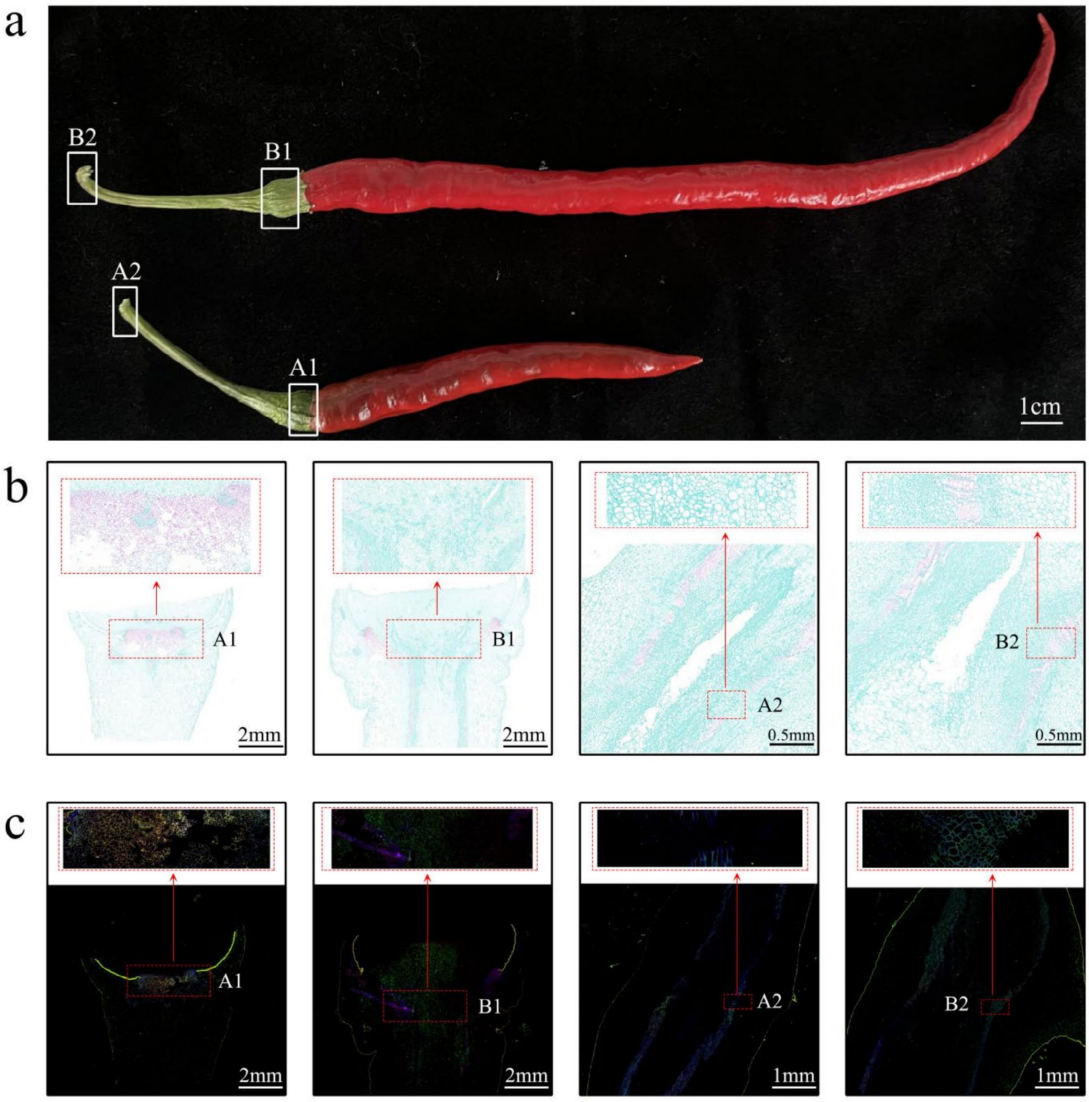
Comparative analysis of abscission zone (AZ) traits in pepper germplasm lines LC‐8 and LC‐17. (a) Phenotypic characterization of AZ detachment: LC‐8 fruit‐end AZ (A1) shows high susceptibility to pedicel abscission, whereas the pedicel‐stem junction (A2) exhibits reduced susceptibility to abscission. In contrast, LC‐17 displays an inverse pattern, with retained fruit‐end AZ (B1: Reduced pedicel abscission susceptibility) and detached shoot‐end AZ (B2: Increased susceptibility to abscission at the pedicel‐stem junction). (b) Safranin O‐fast green‐stained paraffin sections of AZs. LC‐8 A1 displays lignified cells (red) in the abscission layer (AL), contrasting with weakly lignified A2. LC‐17 B2 shows enhanced lignification compared to B1. (c) Fluorol yellow 088 staining reveals suberin accumulation (yellow fluorescence) in AZs.

Two Capsicum germplasm lines were cultivated at the Zunyi Experimental Station (N 27°44′, E 107°12′) of the Pepper Institution, Guizhou Academy of Agricultural Sciences: LC‐8 demonstrates two opposing characteristics: a high susceptibility to pedicel abscission, yet a decreased susceptibility to abscission at the pedicel‐stem junction. Conversely, LC‐17 exhibits a reduced susceptibility to pedicel abscission while displaying an increased susceptibility to abscission at the pedicel‐stem junction under the same conditions. For each germplasm line, four distinct tissues (A1, A2, B1, and B2) were sampled from a total of 100 pepper fruits (Figure 1a). These samples were then divided into two portions: one was preserved in FAA solution at 4°C for subsequent histological analysis, while the other was rapidly frozen in liquid nitrogen and stored at −80°C for later metabolite extraction, transcriptome sequencing, and real‐time PCR analysis.

### 2.2 Observation of Cell Histomorphology

Images were captured to document the macroscopic characteristics. Following this, the tissue surrounding the pedicel abscission zone was sectioned and stained in accordance with established protocols, employing Safranin O‐Fast Green and Fluorol Yellow 088 (Maceda et al. 2021). Each section was examined using light and fluorescence microscopy, and comparative analyses were performed on consecutive sections treated with different staining methods.

### 2.3 RNA‐Seq Analysis

#### 2.3.1 Total RNA Extraction and Quality Assessment

Total RNA was extracted from frozen fruit pedicel abscission tissue using a plant RNA extraction kit. The integrity of the RNA was assessed via agarose gel electrophoresis to detect DNA contamination and confirm sample quality. Subsequently, the quality of the RNA was evaluated using an Agilent 2100 Bioanalyzer.

#### 2.3.2 RNA Library Preparation

cDNA libraries were constructed using the NEBNext Ultra RNA Library Prep Kit for Illumina. Poly(A) mRNA enrichment was performed with Oligo(dT) magnetic beads, followed by lysis in NEB lysis buffer containing divalent cations. The fragmented mRNA was used as a template for first‐strand cDNA synthesis, employing random primers within the M‐MuLV reverse transcriptase system. Subsequent degradation of the RNA strand was carried out by RNase H, facilitating the synthesis of the second‐strand cDNA using a DNA polymerase I system with dNTPs. The resulting double‐stranded cDNA was subjected to end repair, and sequencing adapters were ligated. cDNA fragments of approximately 250–300 bp were selected using AMPure XP beads, followed by PCR amplification and purification. The library concentration was measured with a Qubit 2.0 fluorometer, and the insert size was verified using an Agilent 2100 Bioanalyzer. Quantitative real‐time PCR (qRT‐PCR) was conducted to precisely quantify the DNA concentration. Libraries were pooled in accordance with Illumina HiSeq sequencing specifications to generate 150 bp paired‐end reads.

### 2.4 Metabolomics Analysis

#### 2.4.1 Tissue Sample Extraction

Fruit pedicel abscission tissue (100 mg) was ground to a fine powder in liquid nitrogen. The powdered tissue was suspended in pre‐chilled 80% methanol and vortexed thoroughly. The mixture was incubated on ice for 5 min, followed by centrifugation at 15,000 × *g* and 4°C for 20 min. The supernatant was diluted with LC–MS grade water to achieve a final methanol concentration of 53%, transferred to a new Eppendorf tube, and centrifuged again under the same conditions. The final supernatant was injected into the LC–MS/MS system for analysis.

#### 2.4.2 HPLC–MS/MS Analysis

LC–MS/MS analysis was performed using an ExionLCTM AD system (SCIEX) coupled with a QTRAP 6500+ mass spectrometer (SCIEX). Samples were injected onto an Xselect HSS T3 column (2.1 × 150 mm, 2.5 μm) under a 20‐min linear gradient at a flow rate of 0.4 mL/min in both positive and negative polarity modes. The mobile phases consisted of Eluent A (0.1% formic acid in water) and Eluent B (0.1% formic acid in acetonitrile). The gradient program was set as follows: 2% B for 2 min, 2%–100% B over 15 min, 100% B for 2 min, 100%–2% B over 0.1 min, and 2% B for 2 min.

#### 2.4.3 Metabolite Identification and Quantification

Metabolite detection was conducted in Multiple Reaction Monitoring (MRM) mode based on the Novogene in‐house database. Q3 ions were used for quantification, while Q1, Q3, retention time (RT), declustering potential (DP), and collision energy (CE) were utilized for metabolite identification. HPLC‐MS/MS data files were processed using SCIEX OS Version 1.4 software for peak integration and correction, with parameters set as follows: minimum peak height of 500, signal‐to‐noise ratio of 5, and Gaussian smoothing width of 1. The area of each peak was recorded as the relative content of the corresponding metabolite.

### 2.5 Data Analysis

#### 2.5.1 RNA‐Seq Data

The sequence reads were processed using the Casava software and subsequently converted into FASTQ format. To ensure data integrity, adapter sequences and low‐quality reads were removed, resulting in a dataset of clean reads. Quality metrics, including Q20 and Q30 scores and GC content, were calculated to evaluate the overall quality of the data. Differential expression analysis was conducted using the DESeq2 software package, identifying genes as differentially expressed if they exhibited an adjusted *p*‐value of less than 0.05 and an absolute log_2_ fold change of at least 1. Functional annotation of these differentially expressed genes (DEGs) was carried out through Gene Ontology (GO) and KEGG pathway enrichment analyses, employing the clusterProfiler package.

#### 2.5.2 Metabolomics Data

Metabolomic data were processed using R software (version 4.4.2) with raw intensities normalized by unit variance scaling. Principal component analysis (PCA) was performed to visualize sample clustering patterns. Orthogonal Partial Least Squares‐Discriminant Analysis (OPLS‐DA) models were constructed to identify group‐specific metabolic variations and validated through 200 permutation tests (Q2 intercept < −0.5). Differentially accumulated metabolites (DAMs) were screened using thresholds of variable importance in projection (VIP) ≥ 1, fold change (FC) ≥ 2 or ≤ 0.5, and Student's *t*‐test significance (*p* < 0.05). Biological reproducibility was confirmed by Pearson correlation analysis (R2 > 0.9). Heatmaps (generated using the R “heatmap” package) and boxplots of the top 25 DAMs (selected based on the smallest *p*‐values) were generated to illustrate metabolite profiles.

### 2.6 Integrated Transcriptome and Metabolome Analysis

To enhance our understanding of the relationship between the transcriptome and metabolome, we conducted a mapping of differentially expressed genes (DEGs) and differentially accumulated metabolites (DAMs) onto the KEGG database to identify shared pathway information. Furthermore, pathway analysis was undertaken to examine the DEGs and DAMs associated with lignin biosynthesis pathways and plant hormone signal transduction.

### 2.7 qRT‐PCR Analysis

Nine differentially expressed genes (DEGs) were selected for quantitative real‐time PCR (qRT‐PCR) analysis: LOC107863934 (GID1), LOC107861852 (AUX1), LOC107840223 (CAD), LOC107872884 (PYR), LOC107862076 (4CL), LOC107843777 (TGA), LOC107861980 (CCR), LOC107855732 (EIN3), and LOC107862991 (COMT). For each sample, 10 ng of complementary DNA (cDNA), extracted from the fruit pedicel abscission tissue, was utilized for the analysis. The qRT‐PCR was conducted using the ABI 7500 Real‐Time PCR System (Applied Biosystems, USA) with SYBR Premix Ex Taq (Takara, Japan), adhering to the manufacturer's protocols. EIF5A2 was employed as the internal control gene. Relative expression levels were determined using the 2^−ΔΔCT^ method as described by Livak and Schmittgen (Livak and Schmittgen 2001). Primer sequences employed in this study are detailed in Table S1.

## 3 Results

### 3.1 Morphologic Observation

Figure 1 presents the morphological and histochemical variations observed in the fruit pedicel abscission zones (AZs) of two Capsicum germplasm lines, LC‐8 and LC‐17. Phenotypic analysis (Figure 1a) reveals distinct detachment characteristics: LC‐8 exhibits high susceptibility to pedicel abscission at the fruit‐end AZ (A1) but shows reduced susceptibility to abscission at the pedicel‐stem junction (A2). Conversely, LC‐17 demonstrates reduced susceptibility to pedicel abscission at the fruit‐end AZ (B1) while displaying increased susceptibility to abscission at the pedicel‐stem junction (B2) under similar conditions. Paraffin sections (Figure 1b) indicate dense abscission layers in both AZs of LC‐8 (A1 and A2), with A1 showing enhanced lignification, as evidenced by Safranin O‐Fast Green staining, which correlates with its preferential detachment. Conversely, LC‐17's shoot‐end AZ (B2) displays greater lignin deposition compared to its fruit‐end AZ (B1), consistent with its abscission‐prone phenotype (Figure 1a). Fluorol Yellow 088 staining (Figure 1c) further confirms the spatial patterns of suberin accumulation. Overall, the spatial regulation of lignification and suberin deposition within the AZs is a critical factor in determining the differential abscission susceptibilities.

### 3.2 Transcriptome Sequencing and Annotation

**FIGURE 2:**
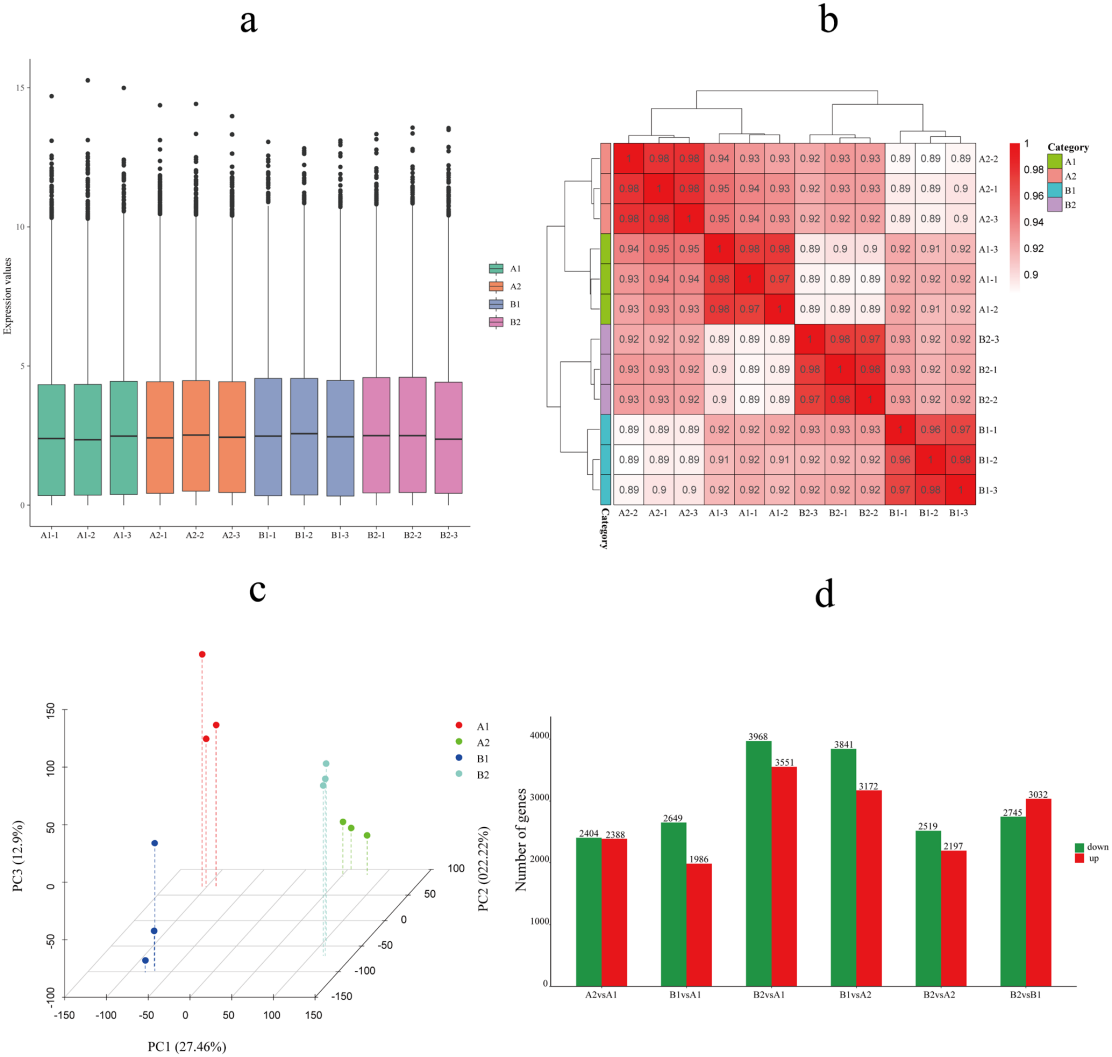
Gene expression analysis. (a) Box figure of FPKM. Horizontal is sample ID and ordinate is log_10_^FPKM^; (b) Pearson correlation between samples; (c) principal component analysis; (d) the number of DEGs in different comparison groups.

In this study, we constructed 12 complementary DNA (cDNA) libraries for Illumina sequencing. From these libraries, we obtained 527,707,656 raw reads and 524,966,780 clean reads. The clean reads from each sample were aligned to the designated reference genome, with alignment rates ranging from 83.8% to 95.7%. The sequencing data exhibited high quality, with Q20 values exceeding 96.9% and Q30 values surpassing 91.4%, and the GC content ranged from 42.7% to 43.0% (Table S2). Furthermore, the gene expression distribution boxplot, Pearson's correlation coefficient (*r*), and principal component analysis (PCA) results indicated a high level of correlation among all samples (Figure 2a–c). These findings confirm that the data quality satisfies the requirements for further analysis.

The analysis encompassed six pairwise comparisons (A1 vs. A2, A1 vs. B1, A1 vs. B2, A2 vs. B1, A2 vs. B2, and B1 vs. B2), revealing 4792, 4635, 7519, 7013, 4716, and 5777 differentially expressed genes (DEGs), respectively. Among these, 2388, 1986, 3551, 3172, 2197, and 3032 DEGs were upregulated, while 2404, 2649, 3968, 3841, 2519, and 2745 were downregulated (Figure 2d; Table S3). The biological functions of the DEGs were assessed using Gene Ontology (GO) annotations. The top 50 significantly enriched GO terms (*p* < 0.05) are presented in Table S3, categorized into biological process (BP), cellular component (CC), and molecular function (MF). In the comparison between A1 and A2, differentially expressed genes (DEGs) were predominantly enriched in the regulation of hormone levels (GO:0010817), thylakoid (GO:0009579), and transferase activity specifically those transferring glycosyl groups (GO:0016757). In the A1 versus B1 comparison, DEGs were primarily enriched in the secondary metabolic process (GO:0019748), thylakoid (GO:0009579), and cofactor binding (GO:0048037). For the A1 versus B2 comparison, DEGs showed significant enrichment in response to water (GO:0009415), thylakoid (GO:0009579), and transferase activity related to glycosyl group transfer (GO:0016757). In the A2 versus B1 comparison, DEGs were mainly enriched in the secondary metabolic process (GO:0019748), intrinsic component of the plasma membrane (GO:0031226), and transcription regulatory region DNA binding (GO:0044212). In the comparison between A2 and B2, DEGs were enriched in the secondary metabolic process (GO:0019748), intrinsic component of the plasma membrane (GO:0031226), and plant‐type cell wall (GO:0009505). Lastly, in the B1 versus B2 comparison, DEGs were predominantly enriched in the regulation of hormone levels (GO:0010817), intrinsic component of the plasma membrane (GO:0031226), and transferase activity involving glycosyl group transfer (GO:0016757).

### 3.3 Kyoto Encyclopedia of Genes and Genomes Enrichment Analysis of the Differentially Expressed Genes

**FIGURE 3:**
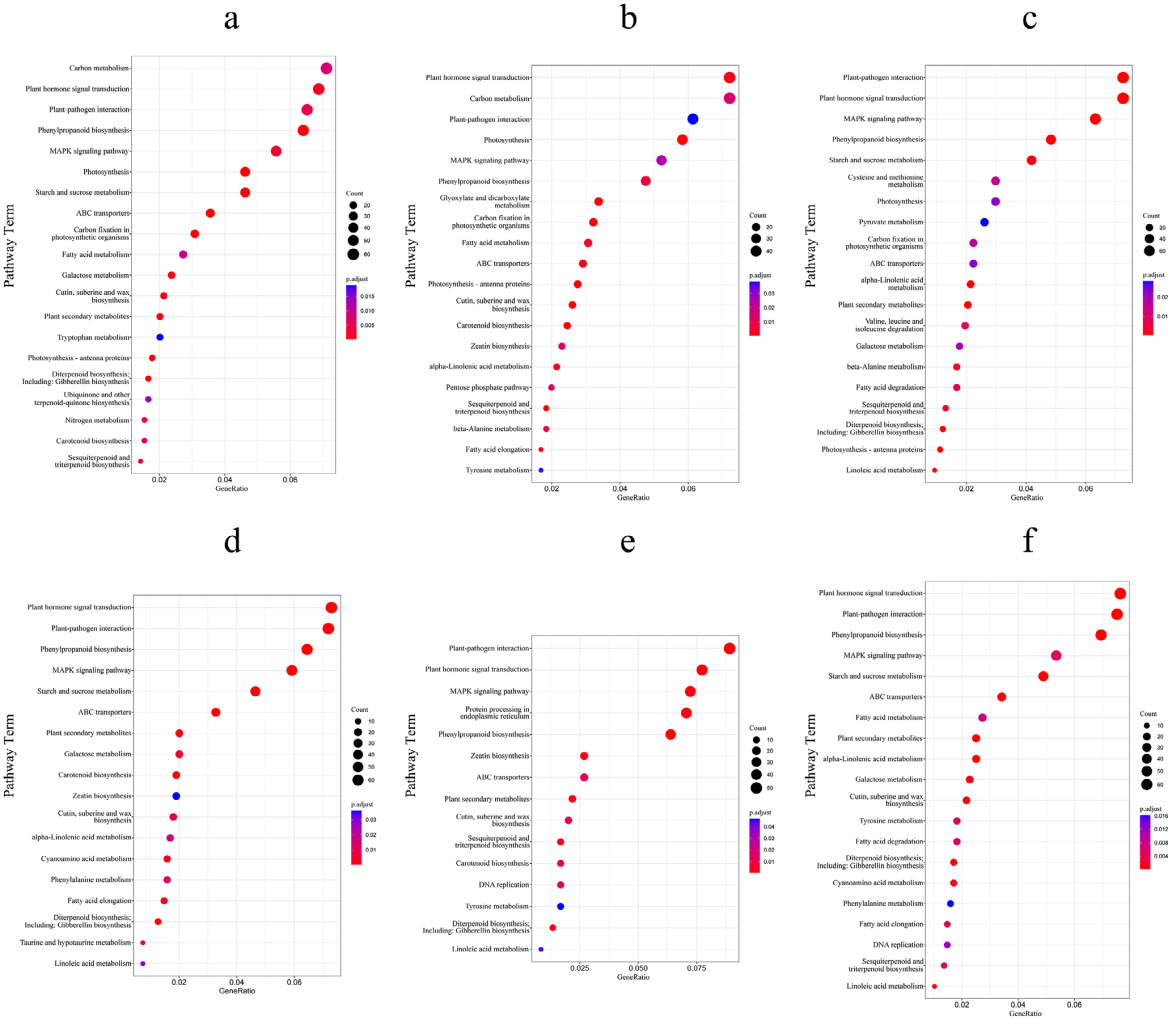
The top 20 enriched KEGG pathways of the DEGs in each comparison: (a) A1 versus A2; (b) A1 versus B1; (c) A1 versus B2; (d) A2 versus B1; (e) A2 versus B2; (f) B1 versus B2.

The top 20 enriched KEGG pathways (*p* < 0.05) are presented in Figure 6. In the comparison between A1 and A2, differentially expressed genes (DEGs) were predominantly enriched in Carbon metabolism, Plant hormone signal transduction, Plant‐pathogen interaction, Phenylpropanoid biosynthesis, and MAPK signaling pathway (Figure 3a). For A1 versus B1, DEGs were mainly enriched in Plant hormone signal transduction, Carbon metabolism, Plant‐pathogen interaction, Photosynthesis, MAPK signaling pathway, and Phenylpropanoid biosynthesis (Figure 3b). In the A1 versus B2 comparison, DEGs showed significant enrichment in Plant‐pathogen interaction, Plant hormone signal transduction, MAPK signaling pathway, Phenylpropanoid biosynthesis, and Starch and sucrose metabolism (Figure 3c). In the A2 versus B1 comparison, DEGs were primarily enriched in Plant hormone signal transduction, Plant‐pathogen interaction, Phenylpropanoid biosynthesis, MAPK signaling pathway, and Starch and sucrose metabolism (Figure 3d). For A2 versus B2, DEGs were enriched in Plant‐pathogen interaction, Plant hormone signal transduction, MAPK signaling pathway, Protein processing in endoplasmic reticulum, and Phenylpropanoid biosynthesis (Figure 3e). Lastly, in the B1 versus B2 comparison, DEGs were enriched in Plant hormone signal transduction, Plant‐pathogen interaction, Phenylpropanoid biosynthesis, MAPK signaling pathway, and Starch and sucrose metabolism (Figure 3f).

### 3.4 Widely Targeted Metabolomics Analysis and Overall Metabolite Identification

**FIGURE 4:**
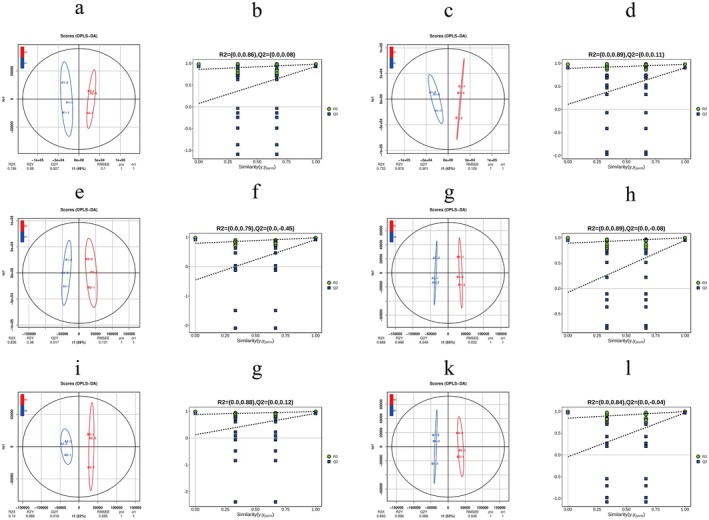
Orthogonal partial least‐squares discriminant (OPLS‐DA) analysis. (a, c, e, g, i, k) Were score plots of the OPLS‐DA model; (b, d, f, h, g, l) were overfitting analyses of the OPLS‐DA model (two hundred permutations). (a, b) A1 versus A2; (c, d) A1 versus B1; (e, f) A1 versus B2; (g, h) A2 versus B1; (i, j) A2 versus B2; (k, l) B1 versus B2.

Orthogonal partial least squares discriminant analysis (OPLS‐DA) effectively discriminated metabolic profiles among experimental groups (Figure 4). The model exhibited robust inter‐group separation across all pairwise comparisons, as evidenced by distinct clustering patterns in score plots (Figure 4a,c,e,g,i,k). Permutation tests with 200 iterations (Figure 4b,d,f,h,j,l) validated model integrity, confirming the absence of overfitting. This rigorous validation procedure demonstrates the high predictive reliability of the metabolomic dataset for downstream functional analyses.

**FIGURE 5:**
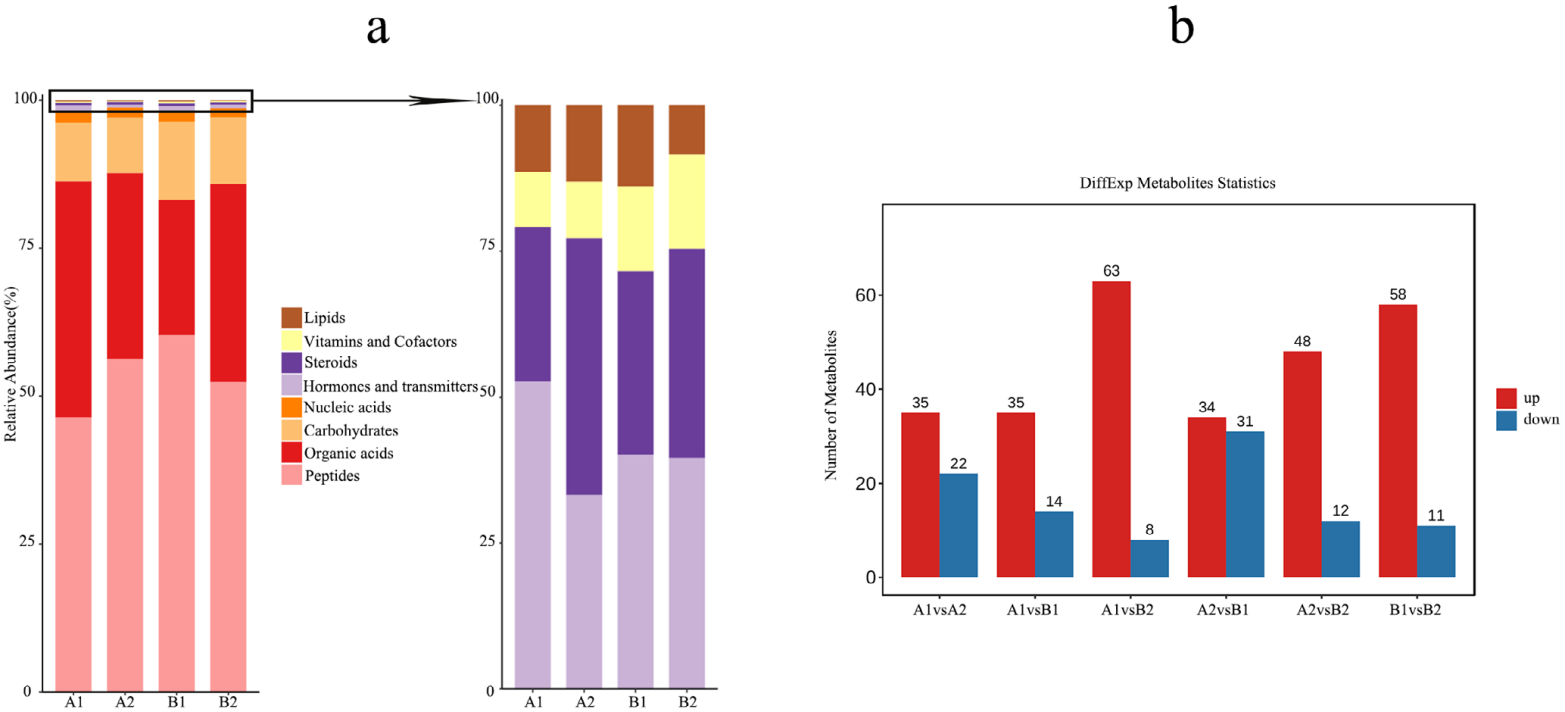
Metabolite analysis of four tissues. (a) Relative content stacked histogram of different metabolite categories. (b) DiffExp metabolites statistics of different tissue types.

Through metabolic profiling, 966 metabolites, including organic acids and their derivatives (322), phenols and their derivatives (157), carbohydrates and their derivatives (97), lipids (66), nucleotides and their derivatives (61), terpenoids (39), alkaloids and derivatives (37), heterocyclic compounds (30), phenylpropanoids and polyketides (26), amines (25), phytohormones (22), vitamins (20), indoles and their derivatives (19), alcohols and polyols (14), benzene and substituted derivatives (13), others (18), were identified, respectively (Figure 5a; Table S4). Details about the identified metabolites are presented in Table S4. Metabolites with a *t*‐test *p* < 0.05 and VIP > 1 were considered to be differentially accumulated metabolites (DAMs) between pairs of samples. As expected, a number of DAMs accumulated between the compared samples, with 57, 49, 71, 65, 60, and 69 DAMs being observed in A1 versus A2, A1 versus B1, A1 versus B2, A2 versus B1, A2 versus B2, and B1 versus B2, respectively (Figure 5b). Among these, 35, 35, 63, 34, 48, and 58 DAMs were upregulated, while 22, 14, 8, 31, 12, and 11 were downregulated.

**FIGURE 6:**
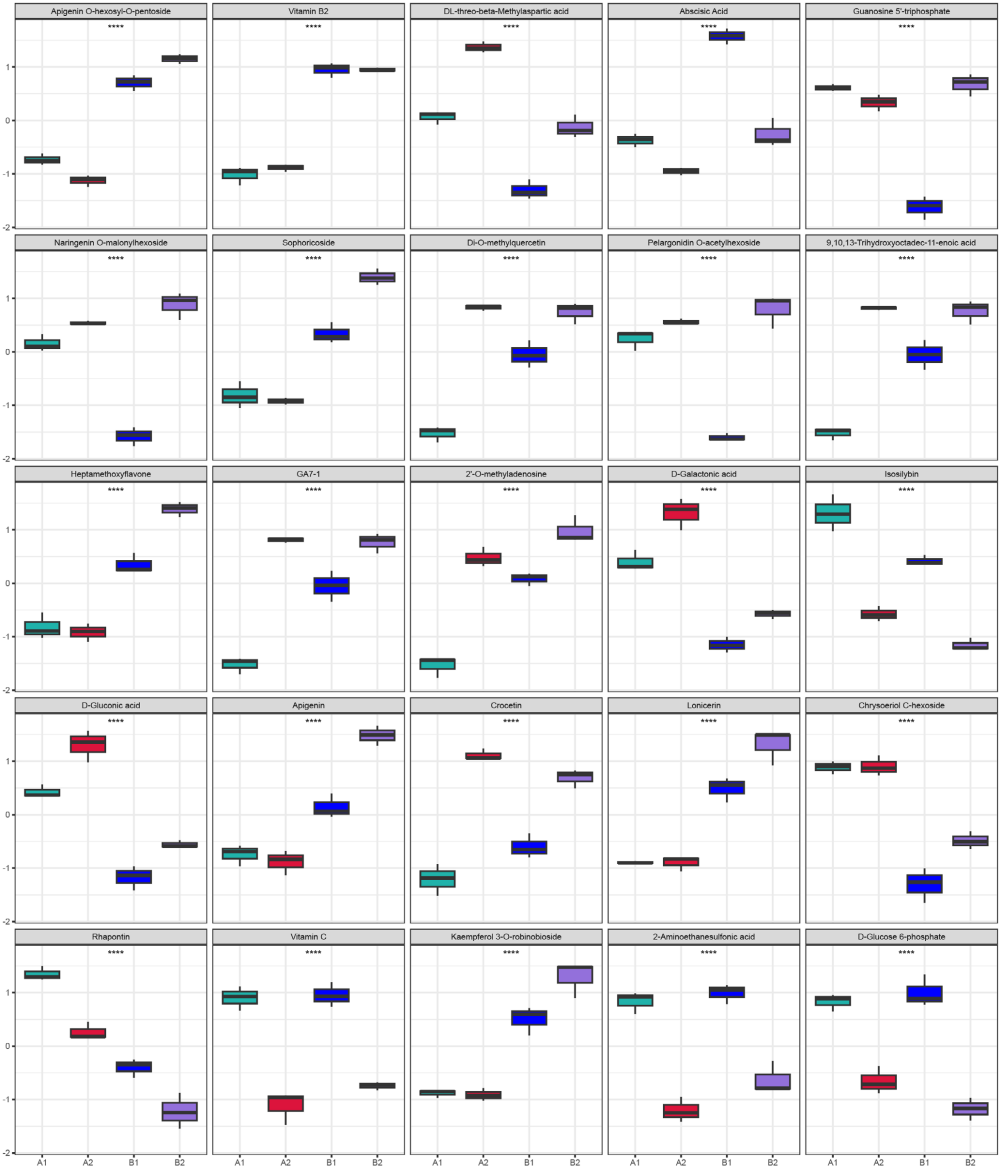
The top 25 differential expression metabolite box line diagram. To intuitively demonstrate the differences in metabolite levels between groups, boxplots were drawn for the top 25 representative metabolites with the smallest *p*‐values identified from univariate statistical analysis. Each box represents one experimental group: A1 (green), A2 (red), B1 (blue), and B2 (purple). Statistical significance was assessed using Student's *t*‐test, and metabolites with **** indicate *p* < 0.0001. Each boxplot is based on *n* = 3 biological replicates per group.

To visually illustrate the intergroup differences of metabolites, we generated box‐and‐whisker plots for the top‐ranked (top 25 metabolites with smallest *p*‐values) representative differential metabolites identified through univariate statistical analysis, as shown in Figure 6. The selected metabolites include: Apigenin O‐hexosyl‐O‐pentoside, Naringenin O‐malonylhexoside, Sophoricoside, Di‐O‐methylquercetin, Heptamethoxyflavone, C‐pentosyl‐C‐hexosyl‐apigenin, Lonicerin, Chrysoeriol C‐hexoside, and Kaempferol 3‐O‐robinobioside from Flavonoids; Vitamin B2 and Vitamin C from Vitamins; DL‐threo‐beta‐Methylaspartic acid from Amino Acid and Derivatives; Abscisic Acid and GA_7‐1_ from Phytohormones; Guanosine 5′‐triphosphate and 2′'‐O‐methyladenosine from Nucleotides and Derivatives; Pelargonidin O‐acetylhexoside from Anthocyanins; 9,10,13‐Trihydroxyoctadec‐11‐enoic acid from Fatty Acyls; D‐Galactonic acid and 2‐Aminoethanesulfonic acid from Organic Acids and Derivatives; Isosilybin from Lignans; D‐Gluconic acid and D‐Glucose 6‐phosphate from Carbohydrates and Derivatives; Crocetin from Terpenoids; and Rhapontin from Phenylpropanoids and Polyketides.

### 3.5 Integrative Analysis of Transcriptomics and Metabolomics

**FIGURE 7:**
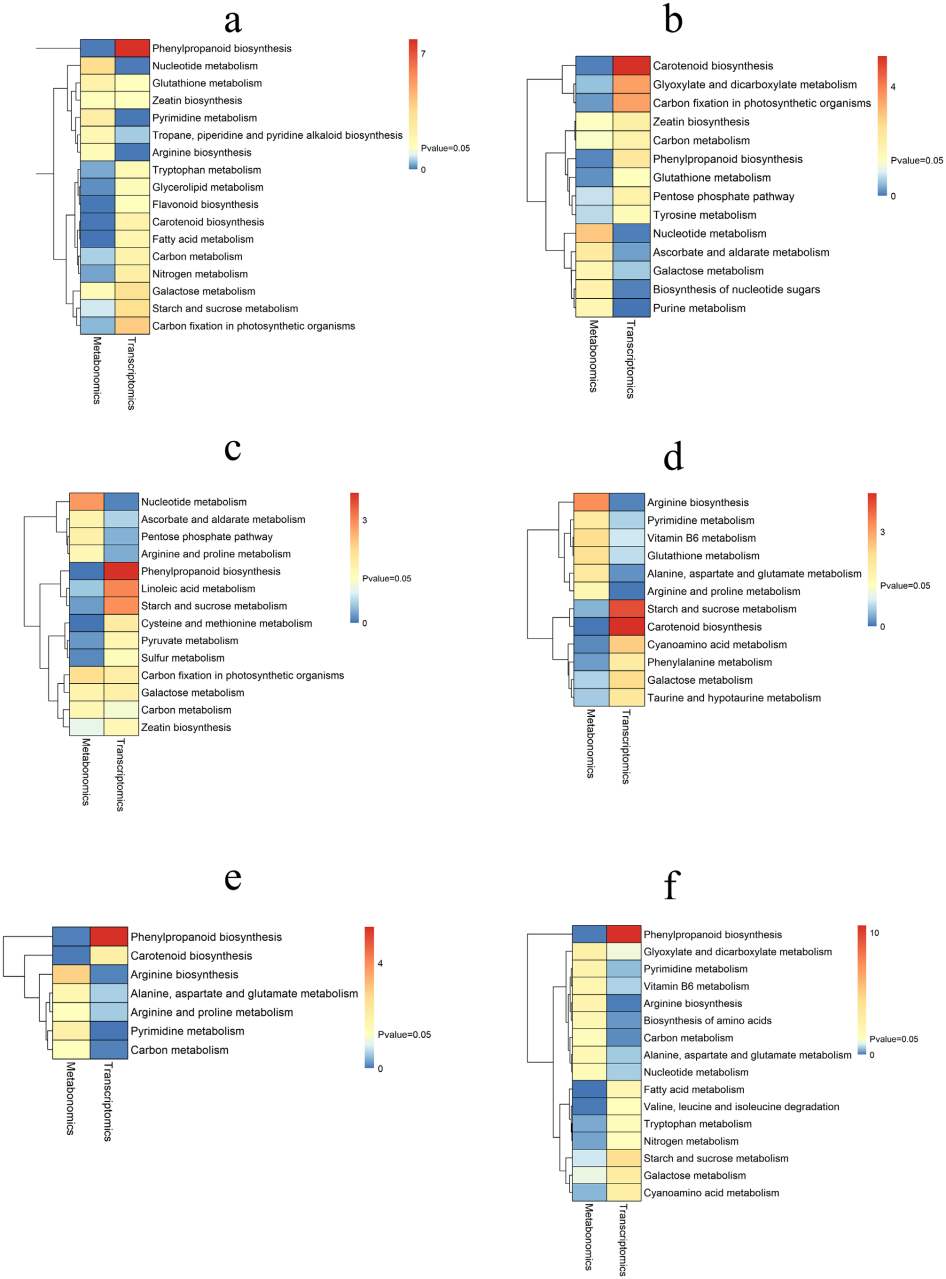
The pvalue_heatmap of KEGG pathways significantly enriched in DEGs and DAMs: (a) A1 versus A2; (b) A1 versus B1; (c) A1 versus B2; (d) A2 versus B1; (e) A2 versus B2; (f) B1 versus B2.

To further investigate the interactive relationships between differentially expressed genes (DEGs) and differentially abundant metabolites (DAMs), we conducted a systematic analysis of shared KEGG pathways showing significant enrichment in either metabolomic or transcriptomic datasets. As illustrated in Figure 7a (A1 vs. A2 comparison), 17 significantly enriched pathways were identified, predominantly featuring “Phenylpropanoid biosynthesis”. In the A1 versus B1 comparison (Figure 7b), 14 enriched pathways were observed, with major representation from “Carotenoid biosynthesis”, “Glyoxylate and dicarboxylate metabolism”, and “Carbon fixation in photosynthetic organisms”, while also retaining the “Phenylpropanoid biosynthesis” pathway. The A1 versus B2 comparison (Figure 7c) revealed 14 enriched pathways, principally including “Phenylpropanoid biosynthesis”, “Linoleic acid metabolism”, and “Starch and sucrose metabolism”. Analysis of the A2 versus B1 group (Figure 7d) demonstrated 12 enriched pathways, prominently featuring “Carotenoid biosynthesis”, “Starch and sucrose metabolism”, and “Phenylalanine metabolism”. In the A2 versus B2 comparison (Figure 7e), 7 enriched pathways were identified, primarily comprising “Phenylpropanoid biosynthesis” and “Carotenoid biosynthesis”. Finally, the B1 versus B2 comparison (Figure 7f) uncovered 16 enriched pathways, with “Phenylpropanoid biosynthesis” being the predominant pathway.

### 3.6 Metabolic Changes and Transcriptomic Regulation Associated With Lignin Synthesis Pathways

**FIGURE 8:**
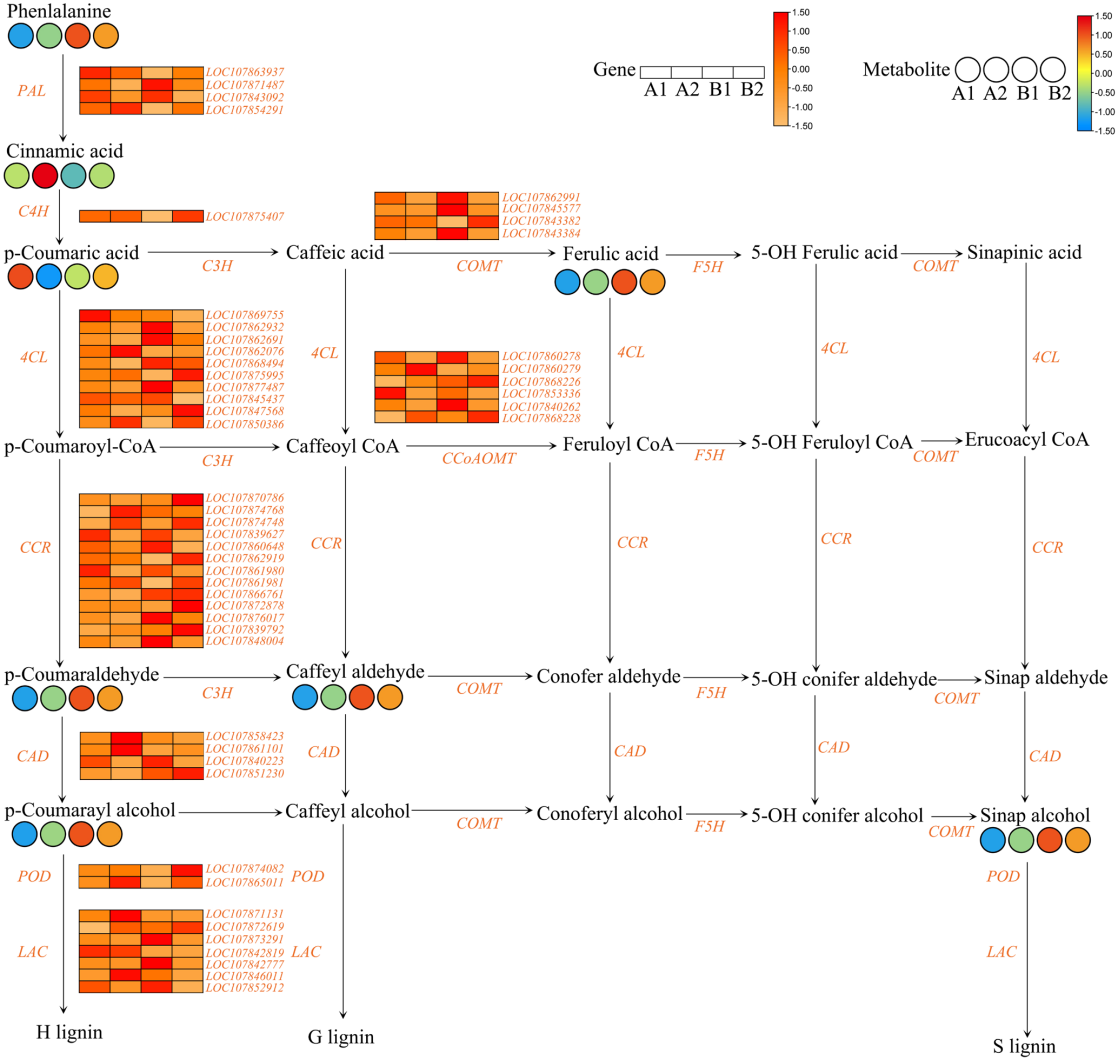
Heatmap of DEGs and DAMs involved in lignin synthesis pathways.

To investigate the roles of genes and metabolites associated with the lignin synthesis pathway in the peduncles of four different pepper cultivars, we analyzed the expression profiles of eight metabolites and 51 genes related to the lignin synthesis pathway (Figure 8). The metabolites included Phenylalanine, Cinnamic acid, p‐Coumaric acid, p‐Coumaroyl‐oA, p‐Coumaraldehyde, p‐Coumaryl alcohol, Caffeyl aldehyde, Ferulic acid, and Sinapyl alcohol. The genes included those encoding 4 *PALs*, 1 *C4H*, 10 *4CLs*, 13 *CCRs*, 4 *CADs*, 2 *PODs*, 7 *LACs*, 4 *COMTs*, and 6 *CCoAOMTs*.

### 3.7 Metabolic Changes and Transcriptomic Regulation Associated With Plant Hormone Signal Transduction

**FIGURE 9:**
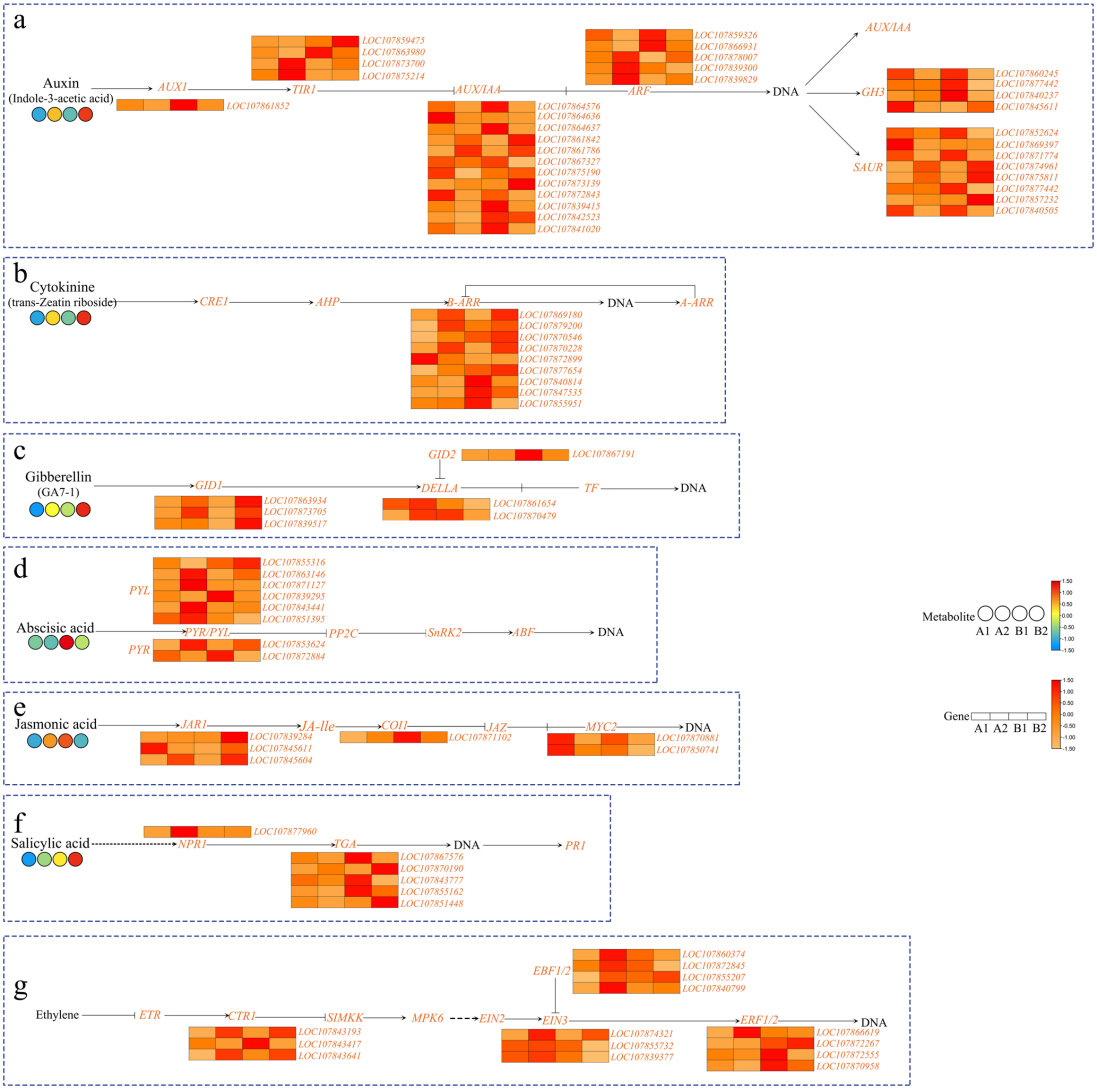
Heatmap of DEGs and DAMs involved in plant hormone signal transduction: a:Auxin; b:Cytokinine; c:Gibberellin; d:Abscisic acid; e:Jasmonic acid; f:Salicylic acid; g:Ethylene.

To investigate the roles of phytohormones in four distinct pepper pedicel tissues, we analyzed the expression profiles of six metabolites associated with phytohormone signaling pathways and 86 differentially expressed genes, as shown in Figure 9. The metabolites comprised: Indole‐3‐acetic acid (Auxin), trans‐Zeatin riboside (Cytokinin), GA_7‐1_ (Gibberellin), Abscisic acid, Jasmonic acid, and Salicylic acid. Distinct expression patterns were observed across signaling pathways: Auxin signaling: 34 genes including 1 AUX1, 4 TIR1, 12 AUX/IAA, 5 ARF, 4 GH3, and 8 SAUR genes. Cytokinin (CTK) signaling: 12 genes comprising 9 B‐ARR and 3 A‐ARR genes. Gibberellin (GA) signaling: 6 genes containing 3 GID1, 1 GID2, and 2 DELLA genes. Abscisic acid (ABA) signaling: 8 genes with 6 PYL and 2 PYR genes. Jasmonic acid (JA) signaling: 6 genes including 1 NPR1 and 2 MYC2 genes. Salicylic acid (SA) signaling: 6 genes consisting of 3 JAR1, 1 COI1, and 5 TGA genes. Additional pathways: 14 genes involving 3 CTR1, 4 EBF1/2, 3 EIN3, and 4 ERF1/2 genes.

### 3.8 Analysis of Gene Expression Through qRT‐PCR

**FIGURE 10:**
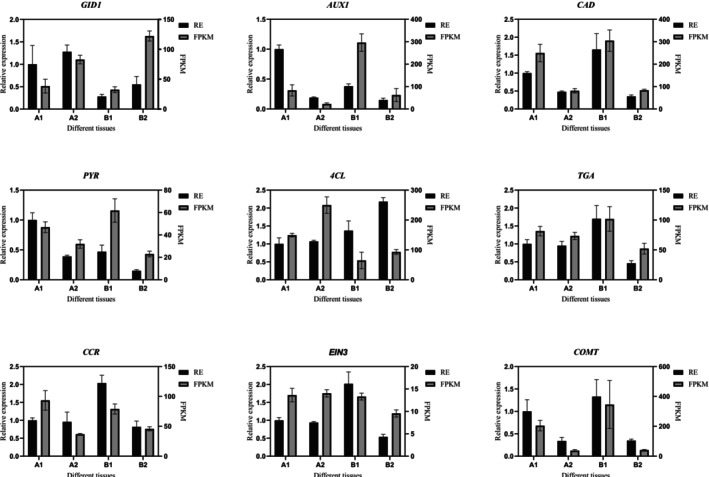
Gene expression validation was conducted using quantitative reverse transcription polymerase chain reaction (qRT‐PCR) analysis. The data presented represent the mean expression values derived from both transcriptomic and qRT‐PCR datasets. Error bars indicate the standard error of the mean (SEM).

To validate the RNA‐seq findings, we conducted quantitative reverse transcription polymerase chain reaction (qRT‐PCR) analysis on nine genes involved in lignin biosynthesis pathways and plant hormone signal transduction. The qRT‐PCR results demonstrate a high degree of concordance with the transcriptomic data (Figure 10).

## 4 Discussion

Previous research has demonstrated that lignification is crucial in floral and fruit abscission processes, with increased lignification accelerating the maturation and lignification of abscission cells (Su et al. 2019; Feng et al. 2021). Our comparative analysis of *Capsicum annuum* germplasm lines LC‐8 and LC‐17 builds upon these findings by identifying key spatial determinants regulating pedicel abscission. Histomorphological analysis reveals cultivar‐specific lignification patterns: LC‐8 exhibits significant lignin accumulation in the distal abscission zone (A1), whereas LC‐17 displays predominant lignification in the proximal separation layer (B2). These results establish a direct relationship between lignin spatial distribution and abscission competence (Figure 1). As a biochemical marker of cellular specialization, lignin biosynthesis plays a pivotal role in the differentiation processes across various tissues, including xylem tracheary elements, pod dehiscence layers, and anther mechanical tissues (Roppolo and Geldner 2012; Hofhuis et al. 2016). Collectively, these findings reveal a spatiotemporal coordination between lignification patterns and abscission susceptibility.

Transcriptome (RNA‐seq) technology is widely recognized as a robust and effective method for quantifying gene expression during plant development. Transcriptome analysis enables the identification of DEGs between varieties and among tissues of the same genotype (Jiang et al. 2025; Shi et al. 2021). In this study, intraspecific comparisons of tissues (A1 vs. A2, B1 vs. B2) detected 4792–5777 DEGs, respectively. Interspecific comparisons across germplasms and tissues (A1 vs. B1, A1 vs. B2, A2 vs. B1, A2 vs. B2) yielded 4635, 7519, 7013, and 4716 DEGs, respectively.

Metabolomic profiling is a well‐established approach for elucidating the dynamic metabolic networks underlying plant developmental processes. For example, multi‐omics integration of transcriptomic and proteomic data revealed coordinated transcriptional and translational reprogramming associated with cell wall metabolism and biosynthetic pathways during pepper fruit development (Liu et al. 2019). Similarly, in the development of apple fruit, a comprehensive analysis of metabolic variations highlighted the interplay between primary and secondary metabolisms, providing a better understanding of fruit quality formation (Xu et al. 2020). Our comprehensive metabolomic profiling identified 966 metabolites spanning 16 categories, including organic acids and derivatives (322), phenolic compounds (157), carbohydrates (97), and lipids (66) (Figure 3a). Univariate statistical analysis (*p* < 0.05, VIP > 1) revealed 268–423 differentially accumulated metabolites (DAMs) across comparison groups (Table S4). Functional annotation of the top‐ranked 25 DAMs (Figure 4) indicated that flavonoid derivatives (e.g., apigenin O‐hexosyl‐O‐pentoside and naringenin O‐malonylhexoside) exert regulatory effects on lignin biosynthesis through dual mechanisms: precursor channeling competition and post‐translational modulation of phenylpropanoid pathway enzymes (Agati et al. 2012; Nabavi et al. 2020). Additionally, abscisic acid (ABA) was found to promote cell wall lignification by activating lignin biosynthesis genes (e.g., CCR, F5H), thereby enhancing plant structural integrity and stress resistance (Rogers and Campbell 2004). In abscission regulation, ABA orchestrates antagonistic processes: (1) promoting cell separation via induction of cellulases and polygalacturonases (Sheldrake 2014), (2) reinforcing abscission zone lignification through cell wall remodeling mediated by peroxidase activity (Lee et al. 2018). Furthermore, ABA accelerates abscission by upregulating ethylene biosynthesis genes, synergizing with ethylene in this process (Wilmowicz et al. 2016). This regulatory mechanism likely involves MYB (e.g., LOC107425254) and NAC transcription factors that bidirectionally control lignification genes and cellulase expression—activating lignification pathways while suppressing cellulase activity to maintain dynamic equilibrium between lignification and cell separation (Gong et al. 2020; Zhang et al. 2021). Notably, gibberellin (GA_7‐1_) antagonizes ABA by suppressing lignification‐related gene expression or signaling pathways, preserving cell wall plasticity to counterbalance ABA‐mediated excessive lignification (Yu et al. 2022).

Integrative transcriptomic‐metabolomic analysis provided two important pathways: phenylpropanoid biosynthesis and plant hormone signal transduction (Figures 7, 8, 9). In the growth and development of plants, there are complex interactions between the biosynthetic pathway of phenylpropanoids, lignin synthesis pathways, and abscission sensitivity. The phenylpropanoid biosynthesis pathway is intrinsically connected to lignin synthesis. In soybeans, the enzyme phenylalanine ammonia‐lyase (PAL) acts as a central component in phenylpropanoid metabolism, with its expression levels demonstrating a significant positive correlation with lignin accumulation (Yuan et al. 2019). Further investigations showed that key enzymes within the phenylpropanoid pathway, such as 4‐coumarate‐CoA ligase (4CL), exerted synergistic regulatory effects on the synthesis of lignin monomers (Chen et al. 2014). Phenylpropanoid metabolites also play a crucial role in the biological regulation of plant organ abscission sensitivity. For instance, grape flower abscission is closely linked to phenylpropanoid metabolites, where flavonoid compounds affect the formation of the abscission zone by modulating auxin polar transport (Domingos et al. 2016). Similarly, in tomatoes, the transcription factor SlBEL11 is involved in fruit retention by inhibiting premature fruit drop through the mediation of flavonoid biosynthesis and regulation of auxin efflux carrier expression (Dong et al. 2024). Plant hormone signaling plays a crucial role in plant growth and development as well as in their response to environmental stress. During the process of plant senescence, hormone signaling also plays an important role. Studies have shown that plant hormones such as ethylene, abscisic acid (ABA), and auxins significantly influence the regulation of organ senescence. First, ethylene is considered a key regulatory factor in plant organ abscission. The activation of the ethylene signaling pathway promotes the expression of abscission‐related genes, which in turn increases the activity of cell wall‐degrading enzymes, ultimately leading to cell separation and organ abscission (Cohen et al. 2014). Additionally, the interaction between ethylene and auxin plays a crucial role in regulating abscission. Studies have found that the polar transport of auxin can inhibit the perception of ethylene, thereby reducing abscission (Kühn et al. 2014). Secondly, abscisic acid (ABA) plays a crucial role in signaling during plants response to adverse stresses. ABA signals can influence the water status and ion balance of plants by regulating the expression of ion channels and aquaporins, indirectly affecting abscission (Zhang et al. 2016). In some cases, enhanced ABA signaling can increase plant sensitivity to abscission (Qin et al. 2019). In addition, the complex network of plant hormone signaling also involves other hormones, such as jasmonate (JA) and salicylic acid (SA). The interaction between jasmonate and other hormones plays a crucial role in balancing plant growth and defense responses (Yang et al. 2019). In some plants, the enhanced jasmonate signal may promote the expression of abscisic acid‐related genes, thereby accelerating organ abscission (Muñoz‐Espinoza et al. 2015). Overall, plant hormone signaling plays a multi‐layered and complex role in regulating organ abscission. The interactions between different hormones and the cross‐regulation of signaling pathways collectively determine the sensitivity of plants to abscission (Chen et al. 2023; Zhao et al. 2024). These studies provide important theoretical foundations for further understanding the molecular mechanisms of plant pedicel abscission susceptibility.

## 5 Conclusions

This study explores the mechanisms underlying the differential susceptibility to abscission in Capsicum fruit pedicels, highlighting that distinct abscission patterns emphasize the specialization of the abscission zone (AZ) through lignification. Transcriptomic analyses across six comparative studies identified between 4635 and 7519 differentially expressed genes (DEGs), which were significantly enriched in key metabolic pathways, including plant hormone signal transduction, phenylpropanoid biosynthesis, and MAPK signaling. A comprehensive metabolomic analysis identified 966 metabolites, with 49 to 71 differentially accumulated metabolites (DAMs) detected in intergroup comparisons. Key metabolites involved in flavonoid, vitamin, and plant hormone pathways were selected for further analysis. Integrated analyses combining transcriptomic and metabolomic data revealed significant enrichment of the phenylpropanoid pathway across multiple comparison groups. Additionally, 51 genes and 8 metabolites related to the lignin biosynthesis pathway, and 86 genes and 6 metabolites involved in plant hormone signal transduction, exhibited differential expression and accumulation. This research provides extensive data for investigating the mechanisms underlying pedicel abscission susceptibility and identifies potential candidate genes and metabolites for future breeding programs aimed at developing pepper cultivars suited for mechanical harvesting.

## Author Contributions

**Lei He:** conceptualization (lead), data curation (equal), formal analysis (equal), funding acquisition (equal), investigation (lead), methodology (equal), project administration (equal), software (lead), supervision (lead), writing – original draft (lead), writing – review and editing (lead). **Xi Yan:** data curation (equal), formal analysis (equal), writing – original draft (equal). **Di Wu:** data curation (equal), methodology (equal), writing – original draft (equal). **Sha Yang:** data curation (equal), formal analysis (equal), writing – original draft (equal). **Wei Lai:** methodology (equal), resources (equal), writing – original draft (equal). **Chongzheng Liu:** methodology (equal), resources (equal), writing – original draft (equal). **Hong Yang:** formal analysis (equal), project administration (equal), resources (equal), supervision (equal), validation (equal), writing – review and editing (equal). **Jianwen He:** formal analysis (equal), funding acquisition (equal), project administration (equal), resources (equal), supervision (equal), validation (equal), writing – review and editing (equal).

## Ethics Statement

The authors have nothing to report.

## Conflicts of Interest

The authors declare no conflicts of interest.

## Supporting information


Table S1.

## Data Availability

All raw data of the RNA‐seq supporting this study were deposited in the NCBI Sequence Read Archive with the ID number PRJNA1233832.
