# Supplementary material for: Integrated Transcriptomic and Metabolomic Profiling Identifies Candidate Genes and Pathways Associated With Pedicel Abscission Susceptibility in Capsicum annuum
Source: Food Sci Nutr. 2025 Jul 24;13(7):e70571. doi: 10.1002/fsn3.70571 (PMC12290222; doi:10.1002/fsn3.70571)

| Table S1. qRT-PCR primers for expression analysis | | |
| --- | --- | --- |
| Gene | Forward primer sequence | Reverse primer sequence |
|  |  |  |
| *GID1* | GGCAGGCAGTAACGAGATCAAC | ACCGACGAAGCATGTTGTAAGC |
| *AUX* | AAACCTCCAACAACTAAAGCACAAG | CCGTCAACTGCCACCTTCAC |
| *CAD* | ACAGTTGGAGTTGGATTGATAGTTGG | TGCCATCAGTGTAGACATCATTGC |
| *PYR* | AACAACAACAACAACAACAACAACAAC | TGGTTATGCTTGAGCTTAGGTTATTGG |
| *4CL* | CATTGTGGACCGATTGAAGGAATTG | ATGTTGGGATGGTTGAGAAGAAGTG |
| *TGA* | TTGGATCAGAAGACATTACGAAGACTC | TGCGATAACTTCAGACGGCTATTC |
| *CCR* | GAAGGACTTGGGCTTGGAGTTTAC | TTGAGTAGGAAGTGGAAGGTGACC |
| *EIN3* | TGGAGAGGCGAATGTGGAAGG | GGTTTGTTTGTTCTTCTGCTTCTCTG |
| *COMT* | ACTCTACCATGACCATGAAGAAGATTC | GTTCCACCACCAACATCAACAATG |
| *EIF5A2* | CCTGTTATCGTGCTACTTTG | GTTTCATTGCCNTGCCAGAT |

| **Table S2.** Quality analysis of RNA sequencing data | | | | | | |
| --- | --- | --- | --- | --- | --- | --- |
| **Sample** | **Raw reads** | **Clean reads** | **Q20(%)** | **Q30(%)** | **GC Content(%)** | **Overall Mapping Rate(%)** |
| A1-1 | 45303944 | 45047428 | 97.4 | 92.4 | 43 | 92.5 |
| A1-2 | 43782936 | 43542106 | 97.4 | 92.6 | 43 | 83.8 |
| A1-3 | 39435252 | 39170392 | 97.2 | 92.1 | 43 | 88.2 |
| A2-1 | 44960896 | 44703276 | 97.3 | 92.2 | 43 | 89 |
| A2-2 | 43468190 | 43244688 | 97.4 | 92.4 | 42.9 | 91.2 |
| A2-3 | 44030188 | 43736054 | 96.9 | 91.5 | 43 | 93 |
| B1-1 | 45217942 | 45046738 | 97.61 | 92.7 | 42.8 | 95.7 |
| B1-2 | 43724602 | 43545124 | 97.4 | 92.5 | 42.8 | 95.7 |
| B1-3 | 39924270 | 39742356 | 97.4 | 92.3 | 42.8 | 95.6 |
| B2-1 | 45521928 | 45285192 | 97.2 | 92.1 | 42.8 | 94.7 |
| B2-2 | 47516418 | 47288754 | 97.3 | 92.1 | 42.8 | 95.2 |
| B2-3 | 44821090 | 44614672 | 97.4 | 92.5 | 42.7 | 94.9 |

| **Table S3-1** GO analysis of DEGs in A1vsA2 | | | | | | | | |
| --- | --- | --- | --- | --- | --- | --- | --- | --- |
| GO_accession | Description | GeneRatio | BgRatio | pvalue | p.adjust | qvalue | Count | Ontology |
| GO:0010817 | regulation of hormone levels | 102/2098 | 482/13776 | 0.000249046 | 0.000249046 | 0.013962555 | 102 | BP |
| GO:0009617 | response to bacterium | 99/2098 | 477/13776 | 0.000618217 | 0.000618217 | 0.026972812 | 99 | BP |
| GO:0019748 | secondary metabolic process | 98/2098 | 382/13776 | 5.85E-08 | 5.85E-08 | 0.0000137 | 98 | BP |
| GO:0071229 | cellular response to acid chemical | 92/2098 | 407/13776 | 0.0000422 | 0.0000422 | 0.004035478 | 92 | BP |
| GO:0022622 | root system development | 90/2098 | 485/13776 | 0.024199029 | 0.024199029 | 0.196309527 | 90 | BP |
| GO:0048364 | root development | 89/2098 | 483/13776 | 2.92E-02 | 2.92E-02 | 2.17E-01 | 89 | BP |
| GO:0009409 | response to cold | 87/2098 | 423/13776 | 1.70E-03 | 1.70E-03 | 5.48E-02 | 87 | BP |
| GO:0009415 | response to water | 86/2098 | 385/13776 | 1.15E-04 | 1.15E-04 | 8.47E-03 | 86 | BP |
| GO:0008610 | lipid biosynthetic process | 86/2098 | 457/13776 | 1.96E-02 | 1.96E-02 | 0.174798899 | 86 | BP |
| GO:0009755 | hormone-mediated signaling pathway | 85/2098 | 437/13776 | 9.02E-03 | 9.02E-03 | 1.17E-01 | 85 | BP |
| GO:1905392 | plant organ morphogenesis | 84/2098 | 441/13776 | 1.57E-02 | 1.57E-02 | 1.62E-01 | 84 | BP |
| GO:0009414 | response to water deprivation | 83/2098 | 377/13776 | 0.000248165 | 0.000248165 | 0.013962555 | 83 | BP |
| GO:0006955 | immune response | 82/2098 | 436/13776 | 2.26E-02 | 2.26E-02 | 0.188790994 | 82 | BP |
| GO:0006812 | cation transport | 81/2098 | 419/13776 | 0.012318116 | 0.012318116 | 0.138282544 | 81 | BP |
| GO:0042742 | defense response to bacterium | 80/2098 | 391/13776 | 0.002950894 | 0.002950894 | 0.076102002 | 80 | BP |
| GO:0009733 | response to auxin | 79/2098 | 326/13776 | 1.13E-05 | 1.13E-05 | 1.46E-03 | 79 | BP |
| GO:0045087 | innate immune response | 78/2098 | 422/13776 | 3.68E-02 | 3.68E-02 | 0.248166214 | 78 | BP |
| GO:0090567 | reproductive shoot system development | 78/2098 | 423/13776 | 0.038553564 | 0.038553564 | 0.256256732 | 78 | BP |
| GO:0048878 | chemical homeostasis | 77/2098 | 388/13776 | 0.00768376 | 0.00768376 | 0.111636714 | 77 | BP |
| GO:0009908 | flower development | 76/2098 | 405/13776 | 0.028570021 | 0.028570021 | 0.214187732 | 76 | BP |
| GO:0032787 | monocarboxylic acid metabolic process | 73/2098 | 380/13776 | 0.019275441 | 0.019275441 | 0.173291954 | 73 | BP |
| GO:0044550 | secondary metabolite biosynthetic process | 72/2098 | 242/13776 | 5.76E-09 | 5.76E-09 | 0.00000186 | 72 | BP |
| GO:0042445 | hormone metabolic process | 72/2098 | 296/13776 | 0.0000238 | 0.0000238 | 0.002458994 | 72 | BP |
| GO:0009620 | response to fungus | 71/2098 | 362/13776 | 0.013262361 | 0.013262361 | 0.143104397 | 71 | BP |
| GO:0009636 | response to toxic substance | 69/2098 | 308/13776 | 0.000479899 | 0.000479899 | 0.02171288 | 69 | BP |
| GO:0006820 | anion transport | 68/2098 | 318/13776 | 0.001911902 | 0.001911902 | 0.058008187 | 68 | BP |
| GO:0048831 | regulation of shoot system development | 67/2098 | 347/13776 | 0.021790439 | 0.021790439 | 0.187947811 | 67 | BP |
| GO:0016049 | cell growth | 66/2098 | 337/13776 | 0.016977296 | 0.016977296 | 0.166359587 | 66 | BP |
| GO:0009611 | response to wounding | 65/2098 | 264/13776 | 0.0000392 | 0.0000392 | 0.003890917 | 65 | BP |
| GO:0009991 | response to extracellular stimulus | 65/2098 | 274/13776 | 1.28E-04 | 1.28E-04 | 0.009158342 | 65 | BP |
| GO:0050832 | defense response to fungus | 63/2098 | 293/13776 | 0.002393703 | 0.002393703 | 0.066113384 | 63 | BP |
| GO:0006720 | isoprenoid metabolic process | 62/2098 | 276/13776 | 0.00084064 | 0.00084064 | 0.032380033 | 62 | BP |
| GO:0046677 | response to antibiotic | 61/2098 | 303/13776 | 0.012139028 | 0.012139028 | 0.138282544 | 61 | BP |
| GO:0071396 | cellular response to lipid | 58/2098 | 244/13776 | 0.000272727 | 0.000272727 | 0.014354067 | 58 | BP |
| GO:0090698 | post-embryonic plant morphogenesis | 58/2098 | 261/13776 | 0.001587077 | 0.001587077 | 0.052324783 | 58 | BP |
| GO:0010015 | root morphogenesis | 58/2098 | 292/13776 | 0.018409625 | 0.018409625 | 0.169562336 | 58 | BP |
| GO:0009579 | thylakoid | 143/2098 | 478/13776 | 9.01E-17 | 9.01E-17 | 1.32E-13 | 143 | CC |
| GO:0009534 | chloroplast thylakoid | 125/2098 | 405/13776 | 6.16E-16 | 6.16E-16 | 3.18E-13 | 125 | CC |
| GO:0042651 | thylakoid membrane | 110/2098 | 336/13776 | 3.75E-16 | 3.75E-16 | 2.42E-13 | 110 | CC |
| GO:0005576 | extracellular region | 109/2098 | 482/13776 | 8.33E-06 | 8.33E-06 | 1.19E-03 | 109 | CC |
| GO:0055035 | plastid thylakoid membrane | 108/2098 | 322/13776 | 1.02E-16 | 1.02E-16 | 1.32E-13 | 108 | CC |
| GO:0009535 | chloroplast thylakoid membrane | 106/2098 | 318/13776 | 3.23E-16 | 3.23E-16 | 2.42E-13 | 106 | CC |
| GO:0031226 | intrinsic component of plasma membrane | 83/2098 | 397/13776 | 0.001315187 | 0.001315187 | 0.045223958 | 83 | CC |
| GO:0048046 | apoplast | 70/2098 | 295/13776 | 0.0000713 | 0.0000713 | 0.006343506 | 70 | CC |
| GO:0009505 | plant-type cell wall | 58/2098 | 262/13776 | 0.001742694 | 0.001742694 | 0.055485378 | 58 | CC |
| GO:0016757 | transferase activity, transferring glycosyl groups | 76/2098 | 346/13776 | 0.000477102 | 0.000477102 | 0.02171288 | 76 | MF |
| GO:0016758 | transferase activity, transferring hexosyl groups | 74/2098 | 305/13776 | 0.0000203 | 0.0000203 | 0.002178829 | 74 | MF |
| GO:0044212 | transcription regulatory region DNA binding | 62/2098 | 330/13776 | 0.043405594 | 0.043405594 | 0.277081048 | 62 | MF |
| GO:0046527 | glucosyltransferase activity | 60/2098 | 197/13776 | 4.17E-08 | 4.17E-08 | 0.0000107 | 60 | MF |
| GO:0008194 | UDP-glycosyltransferase activity | 60/2098 | 221/13776 | 0.00000314 | 0.00000314 | 0.000505989 | 60 | MF |
|  |  |  |  |  |  |  |  |  |
| **Table S3-2** GO analysis of DEGs in A1vsB1 | | | | | | | | |
| GO_accession | Description | GeneRatio | BgRatio | pvalue | p.adjust | qvalue | Count | Ontology |
| GO:0019748 | secondary metabolic process | 102/1658 | 382/13776 | 1.82E-15 | 1.82E-15 | 4.78E-12 | 102 | BP |
| GO:0009415 | response to water | 91/1658 | 385/13776 | 1.01E-10 | 1.01E-10 | 2.93E-08 | 91 | BP |
| GO:0009414 | response to water deprivation | 90/1658 | 377/13776 | 7.30E-11 | 7.30E-11 | 2.39E-08 | 90 | BP |
| GO:0009409 | response to cold | 85/1658 | 423/13776 | 0.00000103 | 0.00000103 | 0.000122956 | 85 | BP |
| GO:0010817 | regulation of hormone levels | 84/1658 | 482/13776 | 0.000268939 | 0.000268939 | 0.013804988 | 84 | BP |
| GO:0071229 | cellular response to acid chemical | 78/1658 | 407/13776 | 1.75E-05 | 1.75E-05 | 1.48E-03 | 78 | BP |
| GO:0008610 | lipid biosynthetic process | 76/1658 | 457/13776 | 1.99E-03 | 1.99E-03 | 5.33E-02 | 76 | BP |
| GO:0032787 | monocarboxylic acid metabolic process | 75/1658 | 380/13776 | 8.70E-06 | 8.70E-06 | 8.14E-04 | 75 | BP |
| GO:0044550 | secondary metabolite biosynthetic process | 72/1658 | 242/13776 | 8.71E-14 | 8.71E-14 | 1.14E-10 | 72 | BP |
| GO:0009617 | response to bacterium | 71/1658 | 477/13776 | 3.31E-02 | 3.31E-02 | 3.65E-01 | 71 | BP |
| GO:0005975 | carbohydrate metabolic process | 70/1658 | 424/13776 | 3.52E-03 | 3.52E-03 | 8.09E-02 | 70 | BP |
| GO:0009755 | hormone-mediated signaling pathway | 67/1658 | 437/13776 | 0.021398726 | 0.021398726 | 0.270626143 | 67 | BP |
| GO:0009908 | flower development | 66/1658 | 405/13776 | 6.10E-03 | 6.10E-03 | 0.11750311 | 66 | BP |
| GO:0090567 | reproductive shoot system development | 66/1658 | 423/13776 | 0.015668489 | 0.015668489 | 0.220529323 | 66 | BP |
| GO:0006979 | response to oxidative stress | 65/1658 | 435/13776 | 0.037353415 | 0.037353415 | 0.379034477 | 65 | BP |
| GO:0042742 | defense response to bacterium | 60/1658 | 391/13776 | 2.78E-02 | 2.78E-02 | 3.16E-01 | 60 | BP |
| GO:0009636 | response to toxic substance | 58/1658 | 308/13776 | 3.25E-04 | 3.25E-04 | 0.015179324 | 58 | BP |
| GO:0042445 | hormone metabolic process | 56/1658 | 296/13776 | 0.000361812 | 0.000361812 | 0.016289696 | 56 | BP |
| GO:0006820 | anion transport | 56/1658 | 318/13776 | 0.002094144 | 0.002094144 | 0.055376249 | 56 | BP |
| GO:0048831 | regulation of shoot system development | 56/1658 | 347/13776 | 0.013157467 | 0.013157467 | 0.202616844 | 56 | BP |
| GO:0009733 | response to auxin | 53/1658 | 326/13776 | 0.013563659 | 0.013563659 | 0.20765048 | 53 | BP |
| GO:0098656 | anion transmembrane transport | 51/1658 | 265/13776 | 0.000429328 | 0.000429328 | 0.017914399 | 51 | BP |
| GO:0006720 | isoprenoid metabolic process | 51/1658 | 276/13776 | 0.001122134 | 0.001122134 | 0.03673698 | 51 | BP |
| GO:0010016 | shoot system morphogenesis | 50/1658 | 234/13776 | 0.0000327 | 0.0000327 | 0.002592285 | 50 | BP |
| GO:0009611 | response to wounding | 48/1658 | 264/13776 | 0.002177841 | 0.002177841 | 0.05644909 | 48 | BP |
| GO:0016051 | carbohydrate biosynthetic process | 47/1658 | 207/13776 | 0.0000108 | 0.0000108 | 0.000973081 | 47 | BP |
| GO:0071396 | cellular response to lipid | 47/1658 | 244/13776 | 0.000692835 | 0.000692835 | 0.026286505 | 47 | BP |
| GO:0090698 | post-embryonic plant morphogenesis | 47/1658 | 261/13776 | 0.002927818 | 0.002927818 | 0.07299732 | 47 | BP |
| GO:0044262 | cellular carbohydrate metabolic process | 47/1658 | 279/13776 | 0.010419526 | 0.010419526 | 0.170482633 | 47 | BP |
| GO:0009735 | response to cytokinin | 46/1658 | 278/13776 | 1.52E-02 | 1.52E-02 | 0.220413446 | 46 | BP |
| GO:0006790 | sulfur compound metabolic process | 46/1658 | 287/13776 | 0.025536244 | 0.025536244 | 0.303869085 | 46 | BP |
| GO:0006631 | fatty acid metabolic process | 42/1658 | 197/13776 | 0.000142464 | 0.000142464 | 0.009096466 | 42 | BP |
| GO:0008299 | isoprenoid biosynthetic process | 41/1658 | 195/13776 | 0.000232458 | 0.000232458 | 0.013229365 | 41 | BP |
| GO:0009579 | thylakoid | 109/1658 | 478/13776 | 1.46E-11 | 1.46E-11 | 5.45E-09 | 109 | CC |
| GO:0009534 | chloroplast thylakoid | 98/1658 | 405/13776 | 4.4E-12 | 4.4E-12 | 1.92E-09 | 98 | CC |
| GO:0042651 | thylakoid membrane | 86/1658 | 336/13776 | 3.88E-12 | 3.88E-12 | 1.92E-09 | 86 | CC |
| GO:0055035 | plastid thylakoid membrane | 84/1658 | 322/13776 | 2.33E-12 | 2.33E-12 | 1.92E-09 | 84 | CC |
| GO:0009535 | chloroplast thylakoid membrane | 83/1658 | 318/13776 | 3.06E-12 | 3.06E-12 | 1.92E-09 | 83 | CC |
| GO:0005576 | extracellular region | 83/1658 | 482/13776 | 0.000431113 | 0.000431113 | 0.017914399 | 83 | CC |
| GO:0031226 | intrinsic component of plasma membrane | 71/1658 | 397/13776 | 3.67E-04 | 3.67E-04 | 1.63E-02 | 71 | CC |
| GO:0048046 | apoplast | 53/1658 | 295/13776 | 1.74E-03 | 1.74E-03 | 0.048048147 | 53 | CC |
| GO:0005887 | integral component of plasma membrane | 49/1658 | 272/13776 | 0.002398282 | 0.002398282 | 0.060955833 | 49 | CC |
| GO:0048037 | cofactor binding | 54/1658 | 225/13776 | 0.00000039 | 0.00000039 | 0.0000567 | 54 | MF |
| GO:0008509 | anion transmembrane transporter activity | 51/1658 | 255/13776 | 0.000163455 | 0.000163455 | 0.009951359 | 51 | MF |
| GO:0016758 | transferase activity, transferring hexosyl groups | 49/1658 | 305/13776 | 0.020908505 | 0.020908505 | 0.267006175 | 49 | MF |
| GO:0016746 | transferase activity, transferring acyl groups | 48/1658 | 242/13776 | 0.000306844 | 0.000306844 | 0.015179324 | 48 | MF |
| GO:0004497 | monooxygenase activity | 47/1658 | 202/13776 | 0.00000538 | 0.00000538 | 0.000521992 | 47 | MF |
| GO:0016705 | oxidoreductase activity, acting on paired donors, with incorporation or reduction of molecular oxygen | 43/1658 | 226/13776 | 0.001472745 | 0.001472745 | 0.043812405 | 43 | MF |
| GO:0008194 | UDP-glycosyltransferase activity | 42/1658 | 221/13776 | 1.70E-03 | 1.70E-03 | 0.047828793 | 42 | MF |
| GO:0016747 | transferase activity, transferring acyl groups other than amino-acyl groups | 41/1658 | 212/13776 | 0.001358463 | 0.001358463 | 0.042847149 | 41 | MF |
|  |  |  |  |  |  |  |  |  |
| **Table S3-3** GO analysis of DEGs in A1vsB2 | | | | | | | | |
| GO_accession | Description | GeneRatio | BgRatio | pvalue | p.adjust | qvalue | Count | Ontology |
| GO:0009415 | response to water | 135/3008 | 385/13776 | 1.12E-09 | 1.12E-09 | 0.00000284 | 135 | BP |
| GO:0009414 | response to water deprivation | 132/3008 | 377/13776 | 1.91E-09 | 1.91E-09 | 0.00000284 | 132 | BP |
| GO:0009617 | response to bacterium | 128/3008 | 477/13776 | 4.92E-03 | 4.92E-03 | 0.112259326 | 128 | BP |
| GO:0010817 | regulation of hormone levels | 128/3008 | 482/13776 | 0.007111005 | 0.007111005 | 0.140964408 | 128 | BP |
| GO:0019748 | secondary metabolic process | 127/3008 | 382/13776 | 0.000000122 | 0.000000122 | 0.0000907 | 127 | BP |
| GO:0006812 | cation transport | 127/3008 | 419/13776 | 2.49E-05 | 2.49E-05 | 6.71E-03 | 127 | BP |
| GO:0042592 | homeostatic process | 127/3008 | 481/13776 | 8.90E-03 | 8.90E-03 | 1.59E-01 | 127 | BP |
| GO:0009755 | hormone-mediated signaling pathway | 126/3008 | 437/13776 | 2.94E-04 | 2.94E-04 | 2.91E-02 | 126 | BP |
| GO:0071229 | cellular response to acid chemical | 125/3008 | 407/13776 | 1.46E-05 | 1.46E-05 | 0.00431888 | 125 | BP |
| GO:0006955 | immune response | 119/3008 | 436/13776 | 3.63E-03 | 3.63E-03 | 9.28E-02 | 119 | BP |
| GO:0006979 | response to oxidative stress | 117/3008 | 435/13776 | 6.44E-03 | 6.44E-03 | 1.35E-01 | 117 | BP |
| GO:0009409 | response to cold | 116/3008 | 423/13776 | 0.003426032 | 0.003426032 | 0.088308515 | 116 | BP |
| GO:0045087 | innate immune response | 115/3008 | 422/13776 | 4.42E-03 | 4.42E-03 | 0.107480115 | 115 | BP |
| GO:0005975 | carbohydrate metabolic process | 114/3008 | 424/13776 | 0.007169933 | 0.007169933 | 0.140964408 | 114 | BP |
| GO:0048878 | chemical homeostasis | 112/3008 | 388/13776 | 0.00059394 | 0.00059394 | 0.035949674 | 112 | BP |
| GO:0009620 | response to fungus | 106/3008 | 362/13776 | 4.72E-04 | 4.72E-04 | 3.18E-02 | 106 | BP |
| GO:0009908 | flower development | 103/3008 | 405/13776 | 4.47E-02 | 4.47E-02 | 0.385406664 | 103 | BP |
| GO:0042742 | defense response to bacterium | 101/3008 | 391/13776 | 0.031935913 | 0.031935913 | 0.305370225 | 101 | BP |
| GO:0009733 | response to auxin | 98/3008 | 326/13776 | 0.000279576 | 0.000279576 | 0.029069923 | 98 | BP |
| GO:0006820 | anion transport | 96/3008 | 318/13776 | 0.000272597 | 0.000272597 | 0.029069923 | 96 | BP |
| GO:0048366 | leaf development | 95/3008 | 362/13776 | 0.024875028 | 0.024875028 | 0.272921238 | 95 | BP |
| GO:0009636 | response to toxic substance | 93/3008 | 308/13776 | 0.000334325 | 0.000334325 | 0.029519379 | 93 | BP |
| GO:0098655 | cation transmembrane transport | 93/3008 | 333/13776 | 0.00474839 | 0.00474839 | 0.111697394 | 93 | BP |
| GO:0044550 | secondary metabolite biosynthetic process | 90/3008 | 242/13776 | 3.05E-08 | 3.05E-08 | 0.0000302 | 90 | BP |
| GO:0009814 | defense response, incompatible interaction | 88/3008 | 318/13776 | 0.007651478 | 0.007651478 | 0.146326398 | 88 | BP |
| GO:0044262 | cellular carbohydrate metabolic process | 85/3008 | 279/13776 | 0.000431139 | 0.000431139 | 0.030665369 | 85 | BP |
| GO:0050832 | defense response to fungus | 85/3008 | 293/13776 | 0.002187648 | 0.002187648 | 0.070719833 | 85 | BP |
| GO:0098656 | anion transmembrane transport | 83/3008 | 265/13776 | 0.000187026 | 0.000187026 | 0.022175389 | 83 | BP |
| GO:0042445 | hormone metabolic process | 82/3008 | 296/13776 | 0.009493582 | 0.009493582 | 0.165535154 | 82 | BP |
| GO:0030001 | metal ion transport | 80/3008 | 247/13776 | 7.07E-05 | 7.07E-05 | 0.01102972 | 80 | BP |
| GO:0046677 | response to antibiotic | 80/3008 | 303/13776 | 0.032357122 | 0.032357122 | 0.308402959 | 80 | BP |
| GO:0009991 | response to extracellular stimulus | 78/3008 | 274/13776 | 0.005491123 | 0.005491123 | 0.12330944 | 78 | BP |
| GO:0071396 | cellular response to lipid | 76/3008 | 244/13776 | 0.000411117 | 0.000411117 | 0.030665369 | 76 | BP |
| GO:0050801 | ion homeostasis | 76/3008 | 270/13776 | 0.008142987 | 0.008142987 | 0.149922538 | 76 | BP |
| GO:0009579 | thylakoid | 130/3008 | 478/13776 | 0.002800938 | 0.002800938 | 0.081397734 | 130 | CC |
| GO:0031226 | intrinsic component of plasma membrane | 126/3008 | 397/13776 | 0.00000222 | 0.00000222 | 0.00109573 | 126 | CC |
| GO:0005576 | extracellular region | 126/3008 | 482/13776 | 0.012666055 | 0.012666055 | 0.187724264 | 126 | CC |
| GO:0009534 | chloroplast thylakoid | 113/3008 | 405/13776 | 0.002077199 | 0.002077199 | 0.070719833 | 113 | CC |
| GO:0042651 | thylakoid membrane | 97/3008 | 336/13776 | 0.001328416 | 0.001328416 | 0.057372319 | 97 | CC |
| GO:0055035 | plastid thylakoid membrane | 93/3008 | 322/13776 | 1.62E-03 | 1.62E-03 | 6.12E-02 | 93 | CC |
| GO:0009535 | chloroplast thylakoid membrane | 91/3008 | 318/13776 | 2.44E-03 | 2.44E-03 | 0.074637042 | 91 | CC |
| GO:0009505 | plant-type cell wall | 88/3008 | 262/13776 | 0.00000653 | 0.00000653 | 0.002419647 | 88 | CC |
| GO:0005887 | integral component of plasma membrane | 85/3008 | 272/13776 | 0.000171424 | 0.000171424 | 0.021172422 | 85 | CC |
| GO:0048046 | apoplast | 83/3008 | 295/13776 | 0.005972174 | 0.005972174 | 0.129217388 | 83 | CC |
| GO:0016757 | transferase activity, transferring glycosyl groups | 99/3008 | 346/13776 | 0.001630478 | 0.001630478 | 0.061178214 | 99 | MF |
| GO:0016758 | transferase activity, transferring hexosyl groups | 91/3008 | 305/13776 | 0.000594268 | 0.000594268 | 0.035949674 | 91 | MF |
| GO:0008324 | cation transmembrane transporter activity | 91/3008 | 306/13776 | 0.000667732 | 0.000667732 | 0.038063445 | 91 | MF |
| GO:0046983 | protein dimerization activity | 82/3008 | 294/13776 | 0.007927986 | 0.007927986 | 0.146876379 | 82 | MF |
| GO:0008509 | anion transmembrane transporter activity | 79/3008 | 255/13776 | 3.85E-04 | 3.85E-04 | 0.030665369 | 79 | MF |
| GO:0022890 | inorganic cation transmembrane transporter activity | 79/3008 | 269/13776 | 0.002133136 | 0.002133136 | 0.070719833 | 79 | MF |
|  |  |  |  |  |  |  |  |  |
| **Table S3-4** GO analysis of DEGs in A2vsB1 | | | | | | | | |
| GO_accession | Description | GeneRatio | BgRatio | pvalue | p.adjust | qvalue | Count | Ontology |
| GO:0019748 | secondary metabolic process | 139/2643 | 382/13776 | 1.05E-15 | 1.05E-15 | 2.43E-12 | 139 | BP |
| GO:0010817 | regulation of hormone levels | 124/2643 | 482/13776 | 0.000204702 | 0.000204702 | 0.01892664 | 124 | BP |
| GO:0009617 | response to bacterium | 116/2643 | 477/13776 | 2.82E-03 | 2.82E-03 | 0.076299175 | 116 | BP |
| GO:0009415 | response to water | 114/2643 | 385/13776 | 0.000000394 | 0.000000394 | 0.000131995 | 114 | BP |
| GO:0009414 | response to water deprivation | 112/2643 | 377/13776 | 0.000000414 | 0.000000414 | 0.000131995 | 112 | BP |
| GO:0009755 | hormone-mediated signaling pathway | 110/2643 | 437/13776 | 1.05E-03 | 1.05E-03 | 4.03E-02 | 110 | BP |
| GO:0006812 | cation transport | 109/2643 | 419/13776 | 3.11E-04 | 3.11E-04 | 2.17E-02 | 109 | BP |
| GO:0006955 | immune response | 109/2643 | 436/13776 | 1.42E-03 | 1.42E-03 | 5.04E-02 | 109 | BP |
| GO:0009409 | response to cold | 108/2643 | 423/13776 | 6.85E-04 | 6.85E-04 | 0.031133645 | 108 | BP |
| GO:1905392 | plant organ morphogenesis | 107/2643 | 441/13776 | 4.32E-03 | 4.32E-03 | 9.63E-02 | 107 | BP |
| GO:0071229 | cellular response to acid chemical | 106/2643 | 407/13776 | 3.57E-04 | 3.57E-04 | 2.30E-02 | 106 | BP |
| GO:0045087 | innate immune response | 105/2643 | 422/13776 | 0.002029455 | 0.002029455 | 0.061254688 | 105 | BP |
| GO:0040007 | growth | 100/2643 | 425/13776 | 1.38E-02 | 1.38E-02 | 0.192532885 | 100 | BP |
| GO:0044550 | secondary metabolite biosynthetic process | 96/2643 | 242/13776 | 8.01E-14 | 8.01E-14 | 7.65E-11 | 96 | BP |
| GO:0090567 | reproductive shoot system development | 96/2643 | 423/13776 | 0.037983881 | 0.037983881 | 0.291213319 | 96 | BP |
| GO:0006820 | anion transport | 94/2643 | 318/13776 | 4.38E-06 | 4.38E-06 | 1.04E-03 | 94 | BP |
| GO:0009908 | flower development | 94/2643 | 405/13776 | 2.33E-02 | 2.33E-02 | 0.222073888 | 94 | BP |
| GO:0042742 | defense response to bacterium | 93/2643 | 391/13776 | 0.012841517 | 0.012841517 | 0.187540321 | 93 | BP |
| GO:0009611 | response to wounding | 92/2643 | 264/13776 | 1.06E-09 | 1.06E-09 | 0.00000061 | 92 | BP |
| GO:0014070 | response to organic cyclic compound | 92/2643 | 393/13776 | 0.01996503 | 0.01996503 | 0.222073888 | 92 | BP |
| GO:0032787 | monocarboxylic acid metabolic process | 91/2643 | 380/13776 | 0.011473087 | 0.011473087 | 0.175631855 | 91 | BP |
| GO:0009620 | response to fungus | 89/2643 | 362/13776 | 0.006012224 | 0.006012224 | 0.116481502 | 89 | BP |
| GO:0009733 | response to auxin | 88/2643 | 326/13776 | 0.000316917 | 0.000316917 | 0.021673744 | 88 | BP |
| GO:0009636 | response to toxic substance | 87/2643 | 308/13776 | 0.000063 | 0.000063 | 0.007531731 | 87 | BP |
| GO:0016049 | cell growth | 87/2643 | 337/13776 | 0.001530373 | 0.001530373 | 0.05043842 | 87 | BP |
| GO:0048589 | developmental growth | 86/2643 | 349/13776 | 0.006418745 | 0.006418745 | 0.119895415 | 86 | BP |
| GO:0048366 | leaf development | 86/2643 | 362/13776 | 0.01670007 | 0.01670007 | 0.20640195 | 86 | BP |
| GO:0046677 | response to antibiotic | 84/2643 | 303/13776 | 0.000167411 | 0.000167411 | 0.018462644 | 84 | BP |
| GO:0009814 | defense response, incompatible interaction | 84/2643 | 318/13776 | 0.000883467 | 0.000883467 | 0.035183674 | 84 | BP |
| GO:0098655 | cation transmembrane transport | 82/2643 | 333/13776 | 7.78E-03 | 7.78E-03 | 0.134701638 | 82 | BP |
| GO:0048831 | regulation of shoot system development | 82/2643 | 347/13776 | 0.021589163 | 0.021589163 | 0.222073888 | 82 | BP |
| GO:0098656 | anion transmembrane transport | 81/2643 | 265/13776 | 0.00000498 | 0.00000498 | 0.001035512 | 81 | BP |
| GO:0042445 | hormone metabolic process | 81/2643 | 296/13776 | 0.000339869 | 0.000339869 | 0.022663483 | 81 | BP |
| GO:0090698 | post-embryonic plant morphogenesis | 80/2643 | 261/13776 | 0.00000506 | 0.00000506 | 0.001035512 | 80 | BP |
| GO:0050832 | defense response to fungus | 79/2643 | 293/13776 | 0.000648828 | 0.000648828 | 0.031133645 | 79 | BP |
| GO:0006720 | isoprenoid metabolic process | 75/2643 | 276/13776 | 0.000688503 | 0.000688503 | 0.031133645 | 75 | BP |
| GO:0060560 | developmental growth involved in morphogenesis | 74/2643 | 285/13776 | 0.002809703 | 0.002809703 | 0.076299175 | 74 | BP |
| GO:0006790 | sulfur compound metabolic process | 73/2643 | 287/13776 | 0.005162386 | 0.005162386 | 0.104242703 | 73 | BP |
| GO:0010243 | response to organonitrogen compound | 71/2643 | 299/13776 | 0.027806383 | 0.027806383 | 0.238716003 | 71 | BP |
| GO:0010016 | shoot system morphogenesis | 70/2643 | 234/13776 | 4.62E-05 | 4.62E-05 | 6.01E-03 | 70 | BP |
| GO:0009991 | response to extracellular stimulus | 70/2643 | 274/13776 | 5.44E-03 | 5.44E-03 | 0.108330895 | 70 | BP |
| GO:0031226 | intrinsic component of plasma membrane | 123/2643 | 397/13776 | 7.89E-09 | 7.89E-09 | 0.00000377 | 123 | CC |
| GO:0005576 | extracellular region | 119/2643 | 482/13776 | 0.001433034 | 0.001433034 | 0.05043267 | 119 | CC |
| GO:0005887 | integral component of plasma membrane | 85/2643 | 272/13776 | 0.00000108 | 0.00000108 | 0.000309602 | 85 | CC |
| GO:0009505 | plant-type cell wall | 77/2643 | 262/13776 | 0.0000388 | 0.0000388 | 0.005376989 | 77 | CC |
| GO:0044212 | transcription regulatory region DNA binding | 93/2643 | 330/13776 | 0.0000394 | 0.0000394 | 0.005376989 | 93 | MF |
| GO:0008509 | anion transmembrane transporter activity | 80/2643 | 255/13776 | 0.00000187 | 0.00000187 | 0.000487429 | 80 | MF |
| GO:0008324 | cation transmembrane transporter activity | 80/2643 | 306/13776 | 0.001585805 | 0.001585805 | 0.051405404 | 80 | MF |
| GO:0016758 | transferase activity, transferring hexosyl groups | 71/2643 | 305/13776 | 4.14E-02 | 4.14E-02 | 0.307166257 | 71 | MF |
| GO:0016705 | oxidoreductase activity, acting on paired donors, with incorporation or reduction of molecular oxygen | 68/2643 | 226/13776 | 0.0000482 | 0.0000482 | 0.0060145 | 68 | MF |
|  |  |  |  |  |  |  |  |  |
| **Table S3-5** GO analysis of DEGs in A2vsB2 | | | | | | | | |
| GO_accession | Description | GeneRatio | BgRatio | pvalue | p.adjust | qvalue | Count | Ontology |
| GO:0019748 | secondary metabolic process | 109/1734 | 382/13776 | 2.72E-17 | 2.72E-17 | 7.45E-14 | 109 | BP |
| GO:0009415 | response to water | 87/1734 | 385/13776 | 2.41E-08 | 2.41E-08 | 0.0000132 | 87 | BP |
| GO:0009414 | response to water deprivation | 86/1734 | 377/13776 | 1.83E-08 | 1.83E-08 | 0.0000125 | 86 | BP |
| GO:0071229 | cellular response to acid chemical | 83/1734 | 407/13776 | 0.00000448 | 0.00000448 | 0.001365867 | 83 | BP |
| GO:0009755 | hormone-mediated signaling pathway | 81/1734 | 437/13776 | 0.000190905 | 0.000190905 | 0.030781206 | 81 | BP |
| GO:0010817 | regulation of hormone levels | 80/1734 | 482/13776 | 5.41E-03 | 5.41E-03 | 2.25E-01 | 80 | BP |
| GO:0044550 | secondary metabolite biosynthetic process | 79/1734 | 242/13776 | 1.99E-16 | 1.99E-16 | 2.73E-13 | 79 | BP |
| GO:1901698 | response to nitrogen compound | 79/1734 | 479/13776 | 6.68E-03 | 6.68E-03 | 2.33E-01 | 79 | BP |
| GO:0009617 | response to bacterium | 74/1734 | 477/13776 | 3.19E-02 | 3.19E-02 | 0.493166352 | 74 | BP |
| GO:0009409 | response to cold | 73/1734 | 423/13776 | 2.90E-03 | 2.90E-03 | 1.68E-01 | 73 | BP |
| GO:0006812 | cation transport | 69/1734 | 419/13776 | 1.11E-02 | 1.11E-02 | 3.07E-01 | 69 | BP |
| GO:0009620 | response to fungus | 68/1734 | 362/13776 | 0.000413023 | 0.000413023 | 0.056605878 | 68 | BP |
| GO:0006955 | immune response | 67/1734 | 436/13776 | 4.69E-02 | 4.69E-02 | 0.618067047 | 67 | BP |
| GO:0032787 | monocarboxylic acid metabolic process | 64/1734 | 380/13776 | 0.00873154 | 0.00873154 | 0.271972836 | 64 | BP |
| GO:0009733 | response to auxin | 63/1734 | 326/13776 | 0.000302754 | 0.000302754 | 0.046103563 | 63 | BP |
| GO:0009611 | response to wounding | 57/1734 | 264/13776 | 2.57E-05 | 2.57E-05 | 6.41E-03 | 57 | BP |
| GO:0010243 | response to organonitrogen compound | 57/1734 | 299/13776 | 8.10E-04 | 8.10E-04 | 0.086836382 | 57 | BP |
| GO:0050832 | defense response to fungus | 56/1734 | 293/13776 | 0.000840263 | 0.000840263 | 0.086836382 | 56 | BP |
| GO:0006820 | anion transport | 56/1734 | 318/13776 | 0.005494708 | 0.005494708 | 0.224807754 | 56 | BP |
| GO:0009636 | response to toxic substance | 55/1734 | 308/13776 | 0.004391714 | 0.004391714 | 0.202329067 | 55 | BP |
| GO:0009753 | response to jasmonic acid | 53/1734 | 252/13776 | 0.000100966 | 0.000100966 | 0.023062866 | 53 | BP |
| GO:0006720 | isoprenoid metabolic process | 52/1734 | 276/13776 | 0.001749235 | 0.001749235 | 0.136035866 | 52 | BP |
| GO:0042445 | hormone metabolic process | 51/1734 | 296/13776 | 0.011774336 | 0.011774336 | 0.31334052 | 51 | BP |
| GO:0098656 | anion transmembrane transport | 49/1734 | 265/13776 | 0.003449725 | 0.003449725 | 0.187611975 | 49 | BP |
| GO:0009991 | response to extracellular stimulus | 49/1734 | 274/13776 | 0.006697007 | 0.006697007 | 0.232827257 | 49 | BP |
| GO:0071396 | cellular response to lipid | 47/1734 | 244/13776 | 0.001786646 | 0.001786646 | 0.136035866 | 47 | BP |
| GO:0030001 | metal ion transport | 46/1734 | 247/13776 | 0.003917021 | 0.003917021 | 0.202329067 | 46 | BP |
| GO:0071669 | plant-type cell wall organization or biogenesis | 44/1734 | 218/13776 | 0.000936475 | 0.000936475 | 0.088514737 | 44 | BP |
| GO:0031668 | cellular response to extracellular stimulus | 44/1734 | 236/13776 | 0.004610372 | 0.004610372 | 0.203826954 | 44 | BP |
| GO:0031667 | response to nutrient levels | 42/1734 | 227/13776 | 6.36E-03 | 6.36E-03 | 0.232827257 | 42 | BP |
| GO:0043161 | proteasome-mediated ubiquitin-dependent protein catabolic process | 42/1734 | 257/13776 | 0.04481226 | 0.04481226 | 0.593394986 | 42 | BP |
| GO:0042594 | response to starvation | 38/1734 | 187/13776 | 0.001772257 | 0.001772257 | 0.136035866 | 38 | BP |
| GO:0016051 | carbohydrate biosynthetic process | 38/1734 | 207/13776 | 0.010332495 | 0.010332495 | 0.291978485 | 38 | BP |
| GO:0031669 | cellular response to nutrient levels | 37/1734 | 190/13776 | 0.004297592 | 0.004297592 | 0.202329067 | 37 | BP |
| GO:0006631 | fatty acid metabolic process | 37/1734 | 197/13776 | 0.007850477 | 0.007850477 | 0.25021593 | 37 | BP |
| GO:0009408 | response to heat | 37/1734 | 218/13776 | 0.034972108 | 0.034972108 | 0.523827261 | 37 | BP |
| GO:0031226 | intrinsic component of plasma membrane | 67/1734 | 397/13776 | 0.007086045 | 0.007086045 | 0.239791849 | 67 | CC |
| GO:0005887 | integral component of plasma membrane | 46/1734 | 272/13776 | 0.021882819 | 0.021882819 | 0.448061547 | 46 | CC |
| GO:0009505 | plant-type cell wall | 45/1734 | 262/13776 | 0.01806221 | 0.01806221 | 0.399269911 | 45 | CC |
| GO:0016757 | transferase activity, transferring glycosyl groups | 55/1734 | 346/13776 | 3.93E-02 | 3.93E-02 | 5.67E-01 | 55 | MF |
| GO:0016758 | transferase activity, transferring hexosyl groups | 54/1734 | 305/13776 | 5.66E-03 | 5.66E-03 | 0.224807754 | 54 | MF |
| GO:0004497 | monooxygenase activity | 53/1734 | 202/13776 | 9.78E-08 | 9.78E-08 | 0.0000447 | 53 | MF |
| GO:0022804 | active transmembrane transporter activity | 53/1734 | 324/13776 | 0.026536992 | 0.026536992 | 0.463307582 | 53 | MF |
| GO:0044212 | transcription regulatory region DNA binding | 53/1734 | 330/13776 | 0.035958539 | 0.035958539 | 0.532779711 | 53 | MF |
| GO:0016705 | oxidoreductase activity, acting on paired donors, with incorporation or reduction of molecular oxygen | 52/1734 | 226/13776 | 0.00000911 | 0.00000911 | 0.002497341 | 52 | MF |
| GO:0008324 | cation transmembrane transporter activity | 49/1734 | 306/13776 | 0.044273146 | 0.044273146 | 0.589102057 | 49 | MF |
| GO:0008509 | anion transmembrane transporter activity | 48/1734 | 255/13776 | 0.002618075 | 0.002618075 | 0.159472905 | 48 | MF |
| GO:0008194 | UDP-glycosyltransferase activity | 41/1734 | 221/13776 | 0.00663728 | 0.00663728 | 0.232827257 | 41 | MF |
| GO:0016709 | oxidoreductase activity, acting on paired donors, with incorporation or reduction of molecular oxygen, NAD(P)H as one donor, and incorporation of one atom of oxygen | 40/1734 | 146/13776 | 1.08E-06 | 1.08E-06 | 0.00037043 | 40 | MF |
| GO:0046527 | glucosyltransferase activity | 40/1734 | 197/13776 | 0.001385763 | 0.001385763 | 0.118701553 | 40 | MF |
|  |  |  |  |  |  |  |  |  |
| **Table S3-6** GO analysis of DEGs in B1vsB2 | | | | | | | | |
| GO_accession | Description | GeneRatio | BgRatio | pvalue | p.adjust | qvalue | Count | Ontology |
| GO:0010817 | regulation of hormone levels | 130/2434 | 482/13776 | 0.000000157 | 0.000000157 | 0.0000236 | 130 | BP |
| GO:0019748 | secondary metabolic process | 120/2434 | 382/13776 | 2.48E-11 | 2.48E-11 | 3.36E-08 | 120 | BP |
| GO:0009617 | response to bacterium | 115/2434 | 477/13776 | 1.85E-04 | 1.85E-04 | 0.006108865 | 115 | BP |
| GO:0006812 | cation transport | 113/2434 | 419/13776 | 0.00000102 | 0.00000102 | 0.0000922 | 113 | BP |
| GO:1905392 | plant organ morphogenesis | 108/2434 | 441/13776 | 0.000152004 | 0.000152004 | 0.005336235 | 108 | BP |
| GO:0022622 | root system development | 108/2434 | 485/13776 | 4.96E-03 | 4.96E-03 | 6.10E-02 | 108 | BP |
| GO:0048364 | root development | 106/2434 | 483/13776 | 8.33E-03 | 8.33E-03 | 8.27E-02 | 106 | BP |
| GO:0042592 | homeostatic process | 105/2434 | 481/13776 | 1.01E-02 | 1.01E-02 | 9.48E-02 | 105 | BP |
| GO:0009755 | hormone-mediated signaling pathway | 104/2434 | 437/13776 | 6.03E-04 | 6.03E-04 | 0.0144169 | 104 | BP |
| GO:0009409 | response to cold | 103/2434 | 423/13776 | 2.68E-04 | 2.68E-04 | 7.99E-03 | 103 | BP |
| GO:0005975 | carbohydrate metabolic process | 102/2434 | 424/13776 | 4.54E-04 | 4.54E-04 | 1.16E-02 | 102 | BP |
| GO:1901698 | response to nitrogen compound | 102/2434 | 479/13776 | 0.021634636 | 0.021634636 | 0.16698303 | 102 | BP |
| GO:0006955 | immune response | 101/2434 | 436/13776 | 1.84E-03 | 1.84E-03 | 0.028569886 | 101 | BP |
| GO:0009415 | response to water | 100/2434 | 385/13776 | 0.0000234 | 0.0000234 | 0.001318344 | 100 | BP |
| GO:0009620 | response to fungus | 99/2434 | 362/13776 | 0.00000245 | 0.00000245 | 0.000200296 | 99 | BP |
| GO:0045087 | innate immune response | 99/2434 | 422/13776 | 1.33E-03 | 1.33E-03 | 2.39E-02 | 99 | BP |
| GO:0090567 | reproductive shoot system development | 98/2434 | 423/13776 | 2.12E-03 | 2.12E-03 | 0.032021195 | 98 | BP |
| GO:0006820 | anion transport | 97/2434 | 318/13776 | 1.11E-08 | 1.11E-08 | 0.000005 | 97 | BP |
| GO:0009414 | response to water deprivation | 97/2434 | 377/13776 | 0.000046 | 0.000046 | 0.002300728 | 97 | BP |
| GO:0008610 | lipid biosynthetic process | 97/2434 | 457/13776 | 0.026637083 | 0.026637083 | 0.196631106 | 97 | BP |
| GO:0009733 | response to auxin | 96/2434 | 326/13776 | 8.86E-08 | 8.86E-08 | 0.0000177 | 96 | BP |
| GO:0032787 | monocarboxylic acid metabolic process | 95/2434 | 380/13776 | 0.000171041 | 0.000171041 | 0.005765629 | 95 | BP |
| GO:0042742 | defense response to bacterium | 95/2434 | 391/13776 | 0.00049288 | 0.00049288 | 0.012223222 | 95 | BP |
| GO:0009908 | flower development | 95/2434 | 405/13776 | 0.001648022 | 0.001648022 | 0.026517041 | 95 | BP |
| GO:0048878 | chemical homeostasis | 92/2434 | 388/13776 | 0.00136518 | 0.00136518 | 0.024030613 | 92 | BP |
| GO:0014070 | response to organic cyclic compound | 92/2434 | 393/13776 | 0.002060344 | 0.002060344 | 0.031288956 | 92 | BP |
| GO:0071229 | cellular response to acid chemical | 91/2434 | 407/13776 | 0.008354511 | 0.008354511 | 0.082723669 | 91 | BP |
| GO:0050832 | defense response to fungus | 88/2434 | 293/13776 | 0.000000115 | 0.000000115 | 0.0000184 | 88 | BP |
| GO:0048366 | leaf development | 87/2434 | 362/13776 | 0.001186386 | 0.001186386 | 0.021930038 | 87 | BP |
| GO:0044550 | secondary metabolite biosynthetic process | 85/2434 | 242/13776 | 4.25E-11 | 4.25E-11 | 3.83E-08 | 85 | BP |
| GO:0042445 | hormone metabolic process | 84/2434 | 296/13776 | 0.00000293 | 0.00000293 | 0.000220095 | 84 | BP |
| GO:0098655 | cation transmembrane transport | 84/2434 | 333/13776 | 0.000291352 | 0.000291352 | 0.008253152 | 84 | BP |
| GO:0090698 | post-embryonic plant morphogenesis | 83/2434 | 261/13776 | 0.000000016 | 0.000000016 | 0.00000619 | 83 | BP |
| GO:0098656 | anion transmembrane transport | 83/2434 | 265/13776 | 3.45E-08 | 3.45E-08 | 0.0000113 | 83 | BP |
| GO:0009611 | response to wounding | 80/2434 | 264/13776 | 0.000000287 | 0.000000287 | 0.0000369 | 80 | BP |
| GO:0048831 | regulation of shoot system development | 77/2434 | 347/13776 | 0.017095584 | 0.017095584 | 0.143515722 | 77 | BP |
| GO:0046677 | response to antibiotic | 76/2434 | 303/13776 | 0.000660088 | 0.000660088 | 0.015279761 | 76 | BP |
| GO:0006720 | isoprenoid metabolic process | 75/2434 | 276/13776 | 0.0000495 | 0.0000495 | 0.002388133 | 75 | BP |
| GO:0009753 | response to jasmonic acid | 74/2434 | 252/13776 | 0.00000291 | 0.00000291 | 0.000220095 | 74 | BP |
| GO:0031226 | intrinsic component of plasma membrane | 125/2434 | 397/13776 | 7.95E-12 | 7.95E-12 | 2.15E-08 | 125 | CC |
| GO:0005576 | extracellular region | 105/2434 | 482/13776 | 1.07E-02 | 1.07E-02 | 0.097885718 | 105 | CC |
| GO:0009505 | plant-type cell wall | 82/2434 | 262/13776 | 4.32E-08 | 4.32E-08 | 0.0000117 | 82 | CC |
| GO:0005887 | integral component of plasma membrane | 82/2434 | 272/13776 | 0.00000026 | 0.00000026 | 0.0000352 | 82 | CC |
| GO:0016757 | transferase activity, transferring glycosyl groups | 89/2434 | 346/13776 | 0.000094 | 0.000094 | 0.003682678 | 89 | MF |
| GO:0044212 | transcription regulatory region DNA binding | 84/2434 | 330/13776 | 0.000212254 | 0.000212254 | 0.006830435 | 84 | MF |
| GO:0008324 | cation transmembrane transporter activity | 83/2434 | 306/13776 | 0.0000216 | 0.0000216 | 0.001243229 | 83 | MF |
| GO:0016758 | transferase activity, transferring hexosyl groups | 81/2434 | 305/13776 | 0.0000602 | 0.0000602 | 0.002854264 | 81 | MF |
| GO:0008509 | anion transmembrane transporter activity | 80/2434 | 255/13776 | 5.62E-08 | 5.62E-08 | 0.0000138 | 80 | MF |
| GO:0046983 | protein dimerization activity | 77/2434 | 294/13776 | 1.47E-04 | 1.47E-04 | 0.005299893 | 77 | MF |
| GO:0022804 | active transmembrane transporter activity | 75/2434 | 324/13776 | 0.006730391 | 0.006730391 | 0.07106762 | 75 | MF |


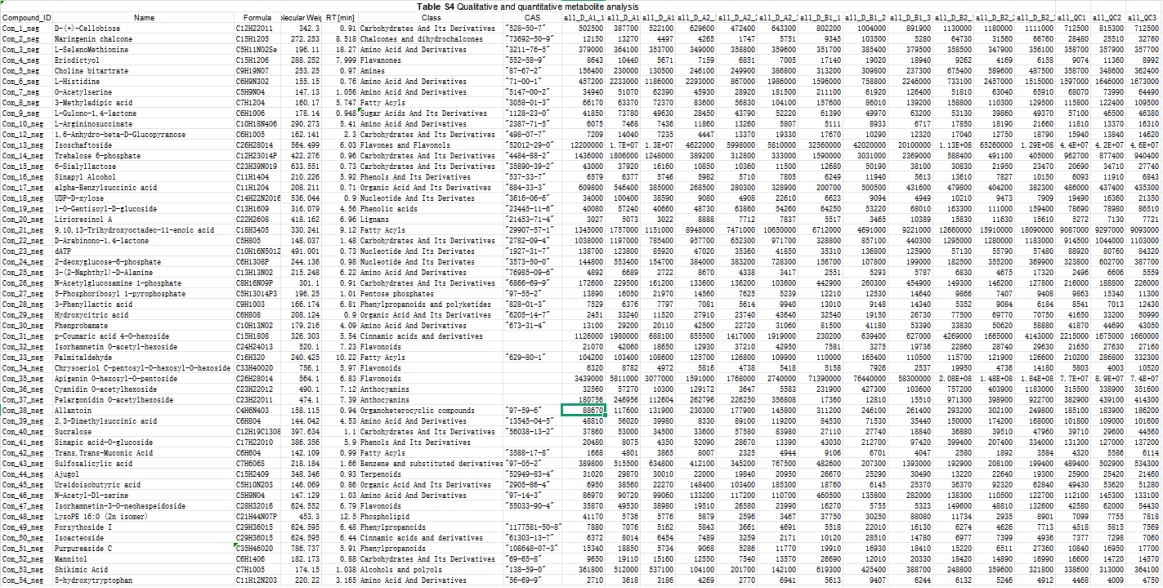

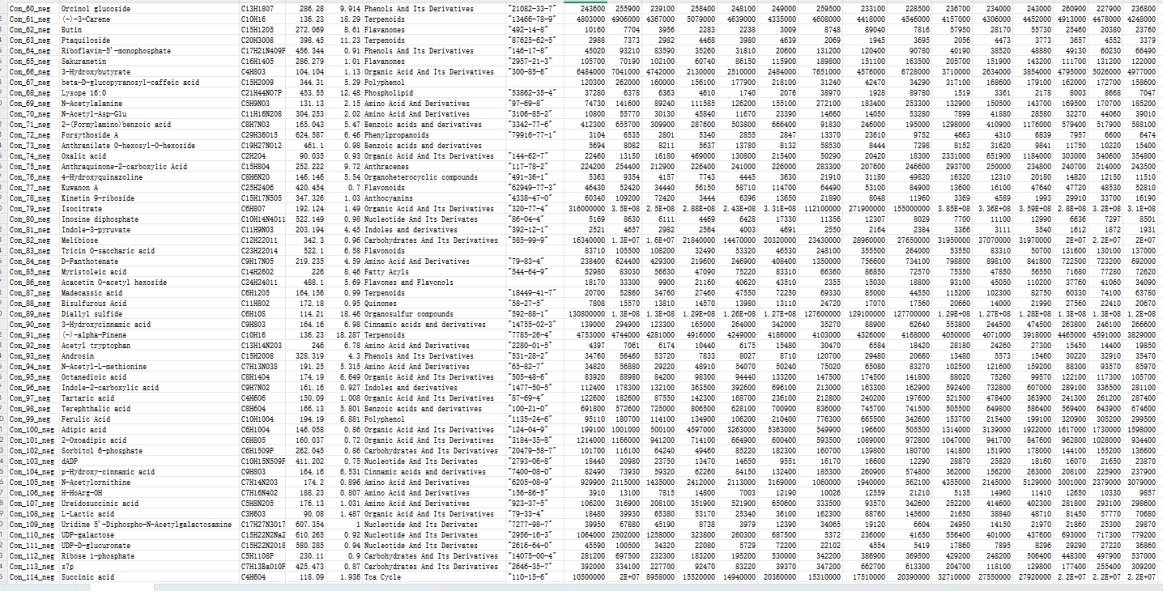

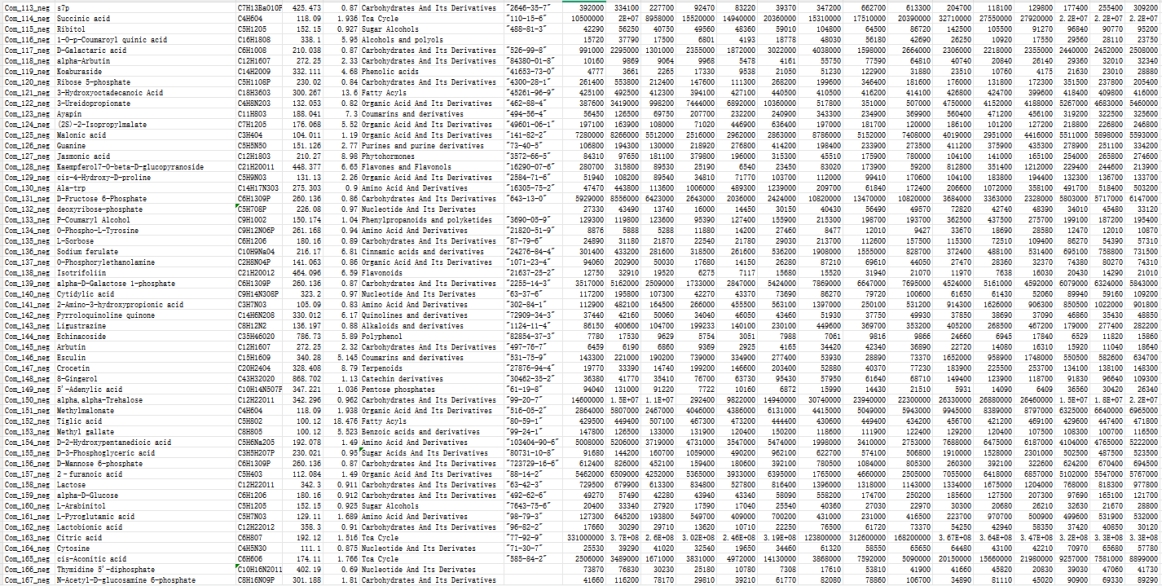

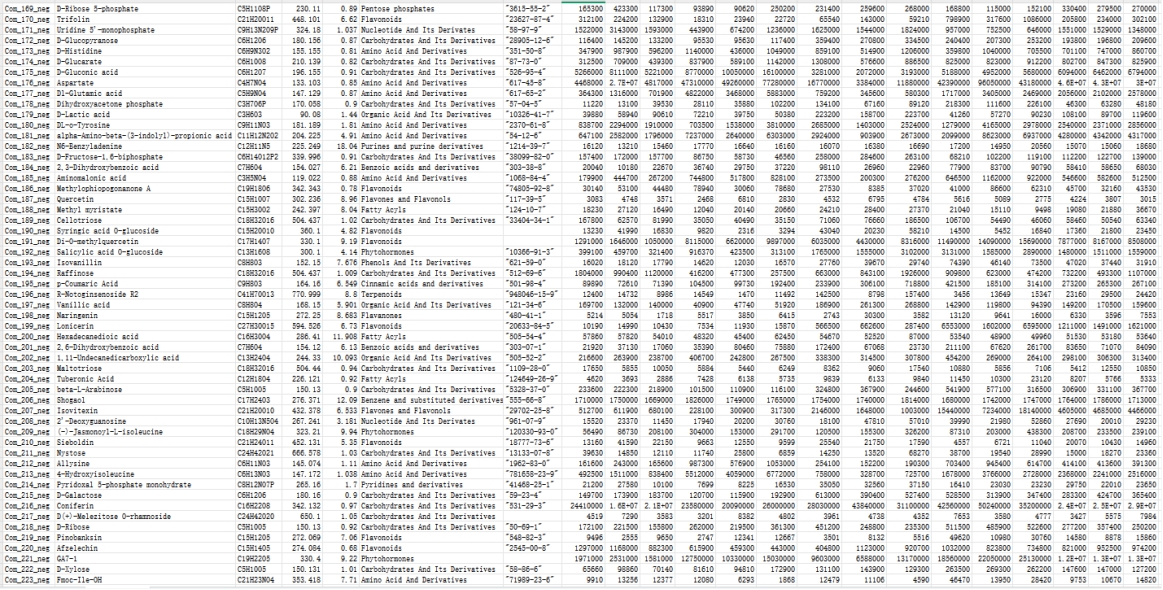

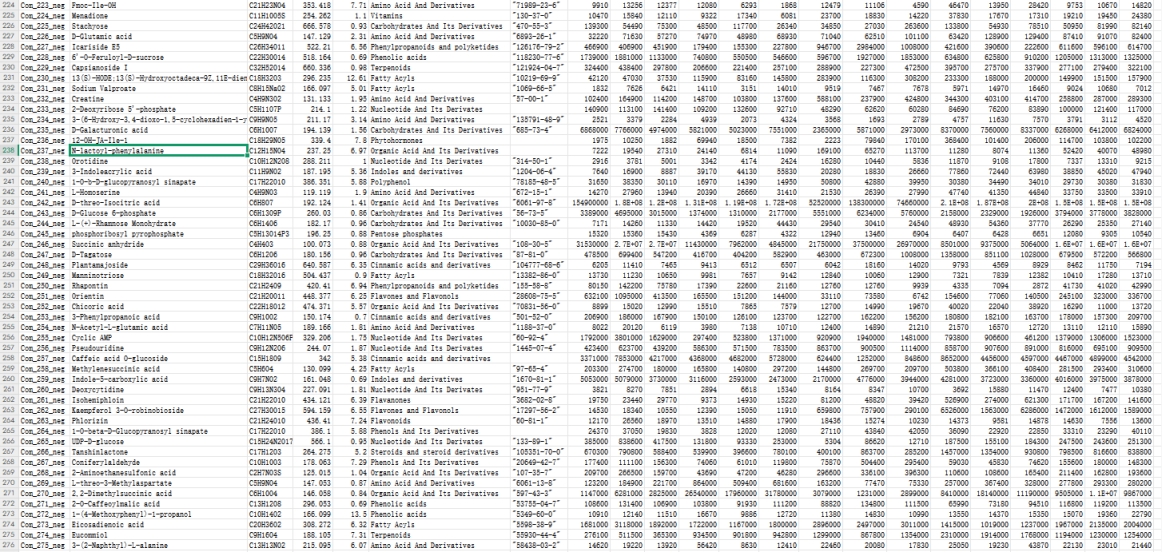

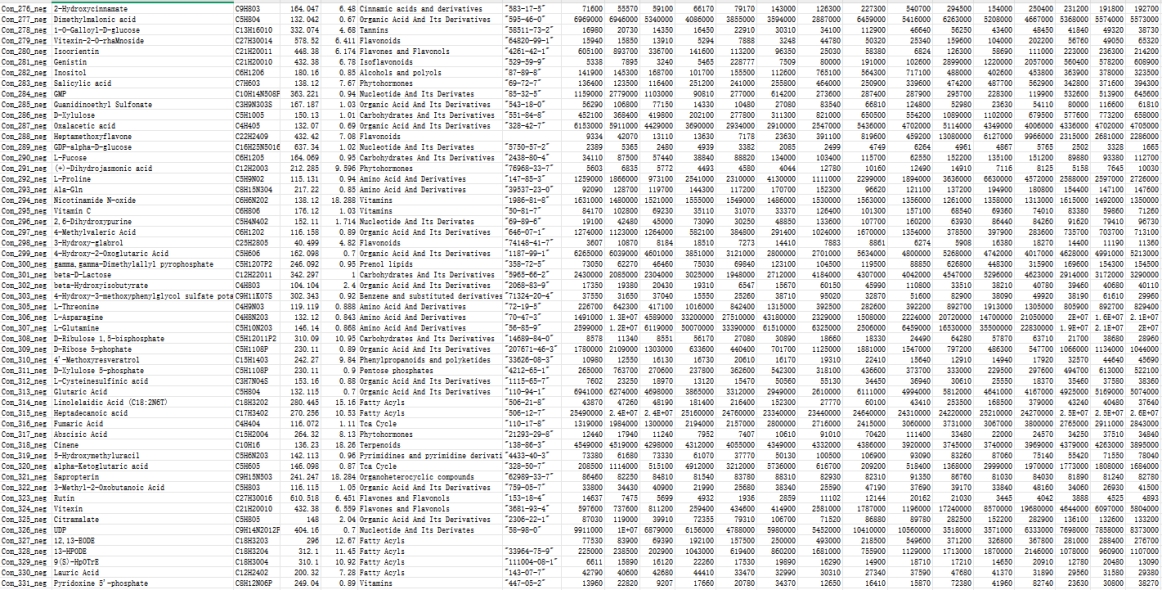

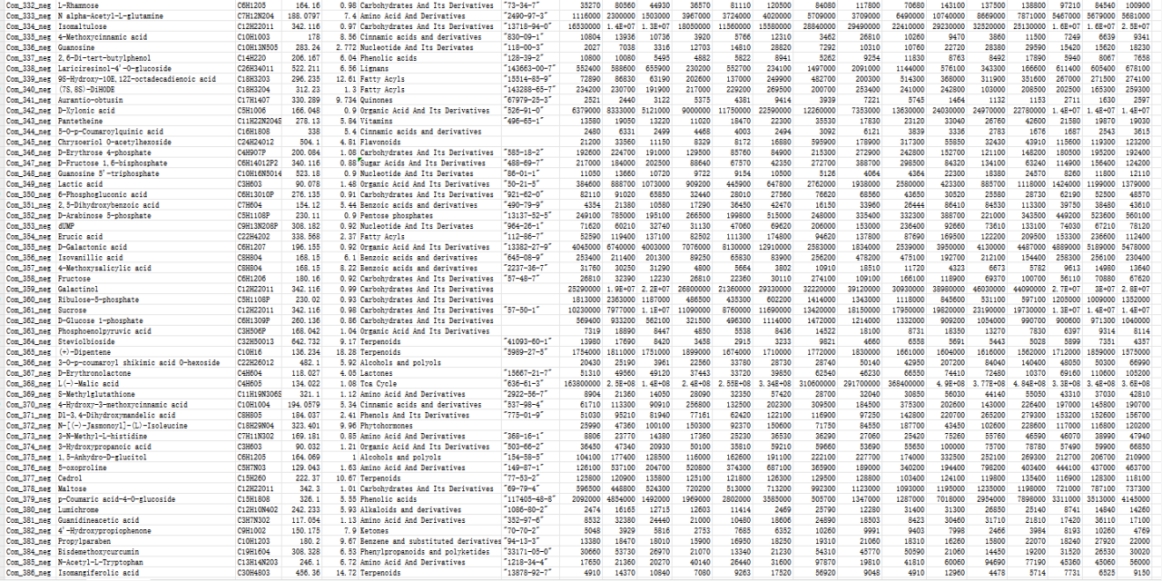

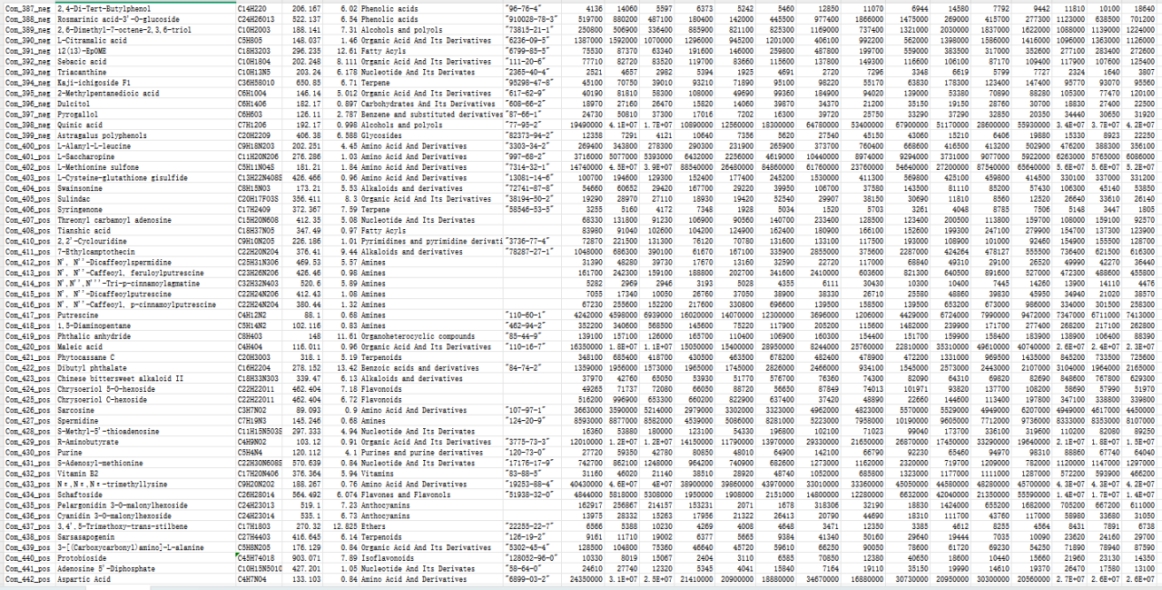

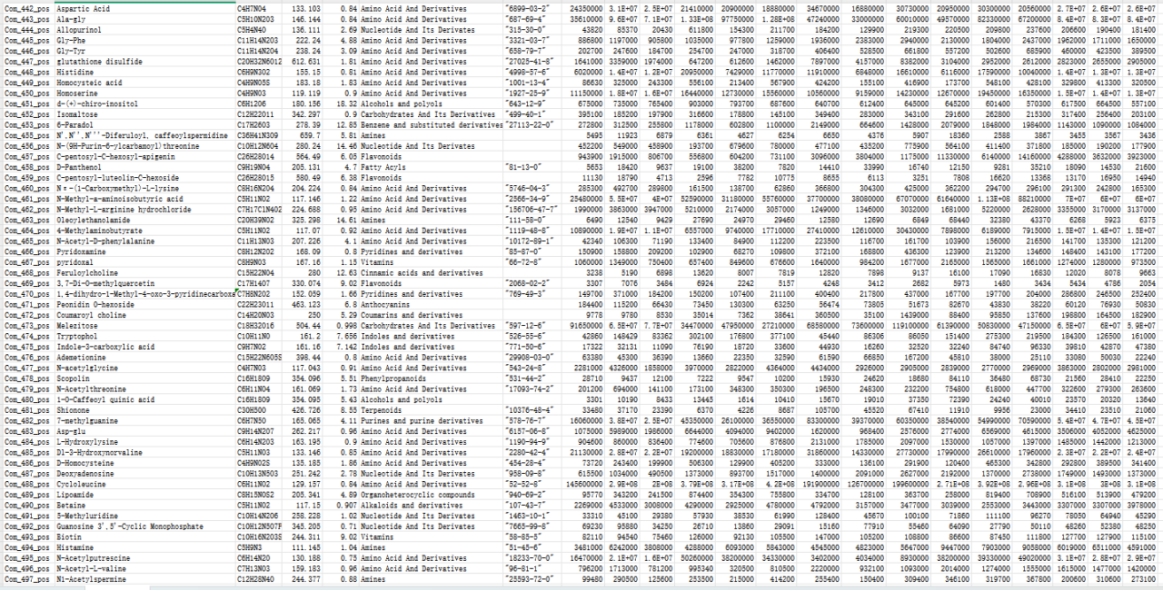

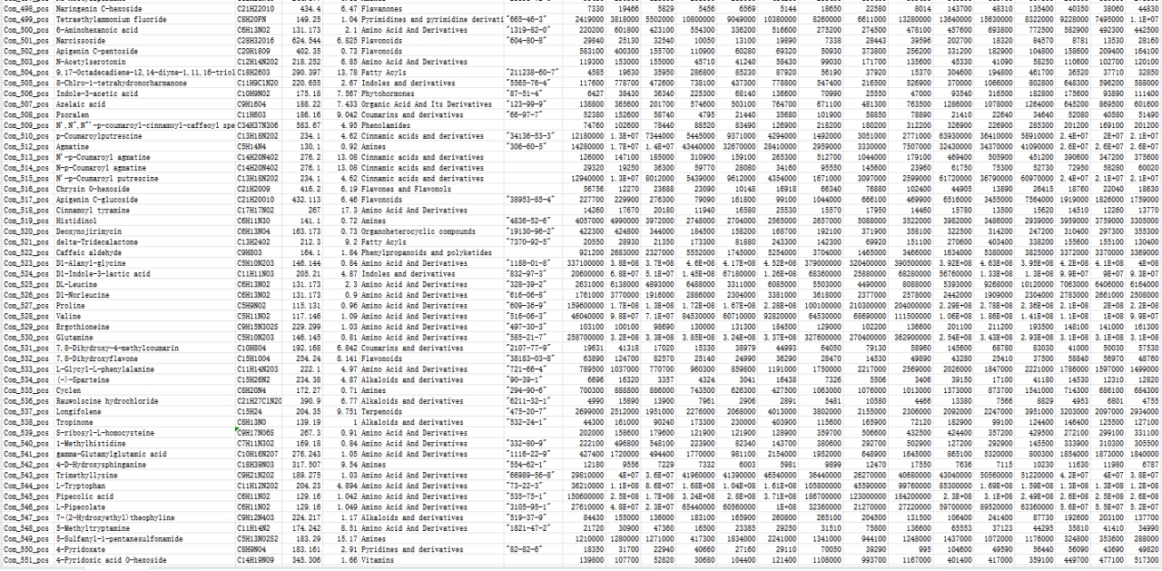

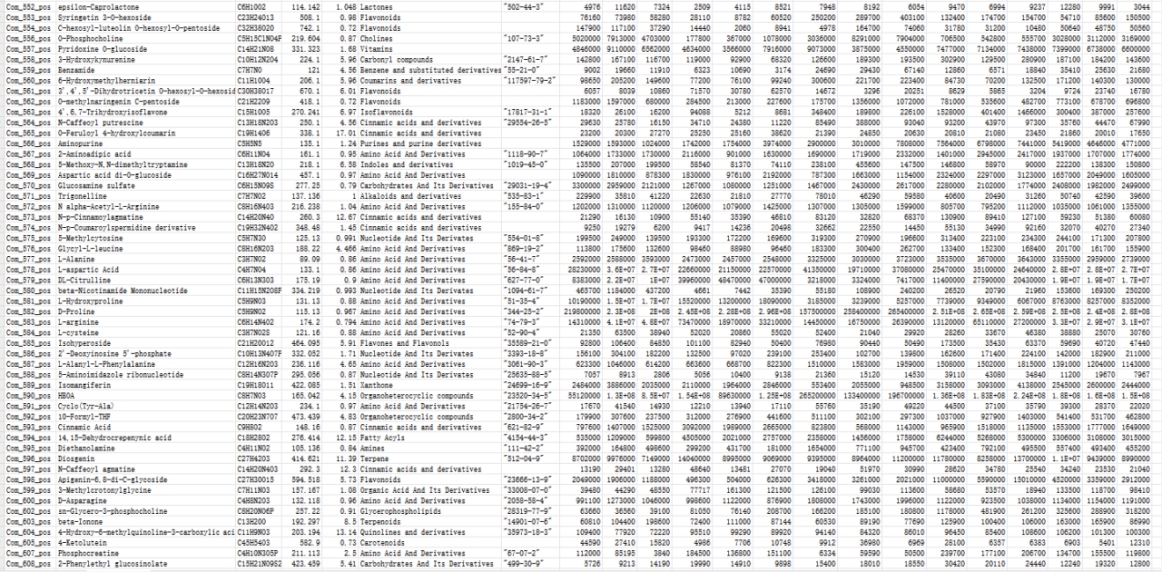

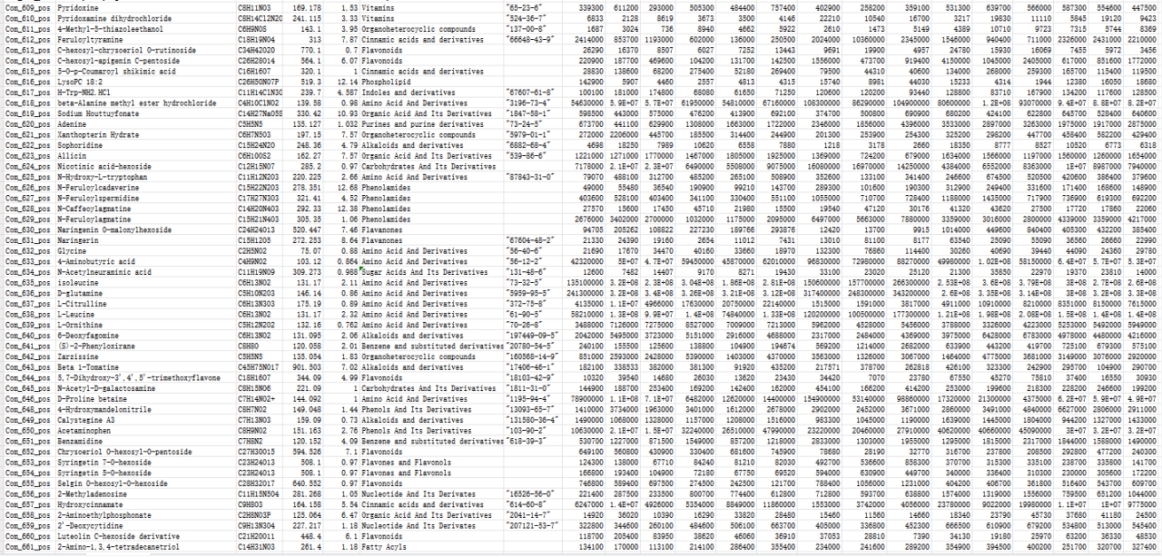

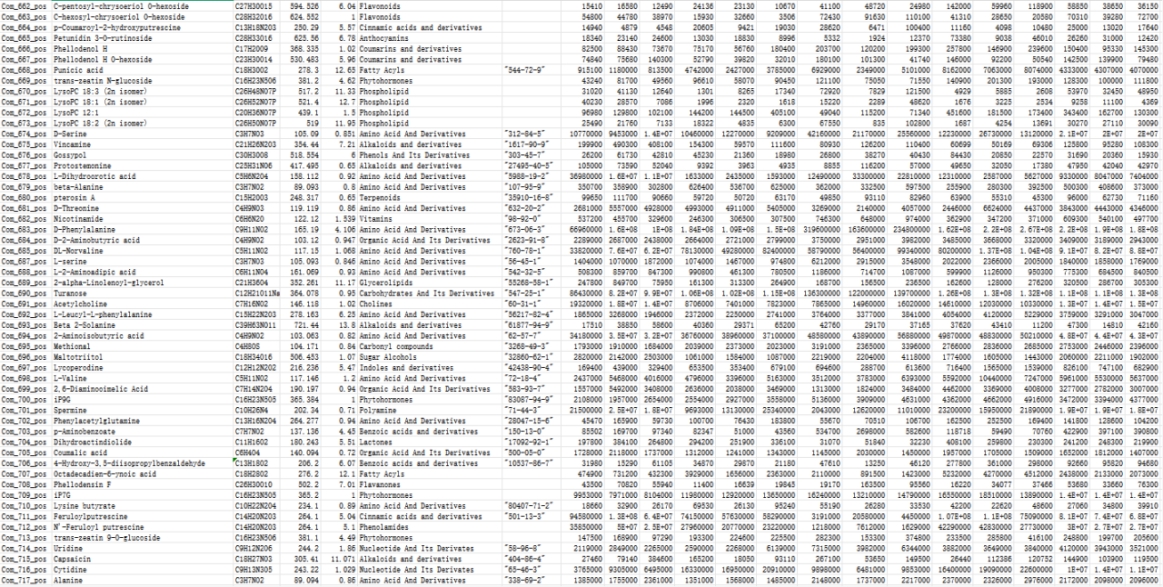

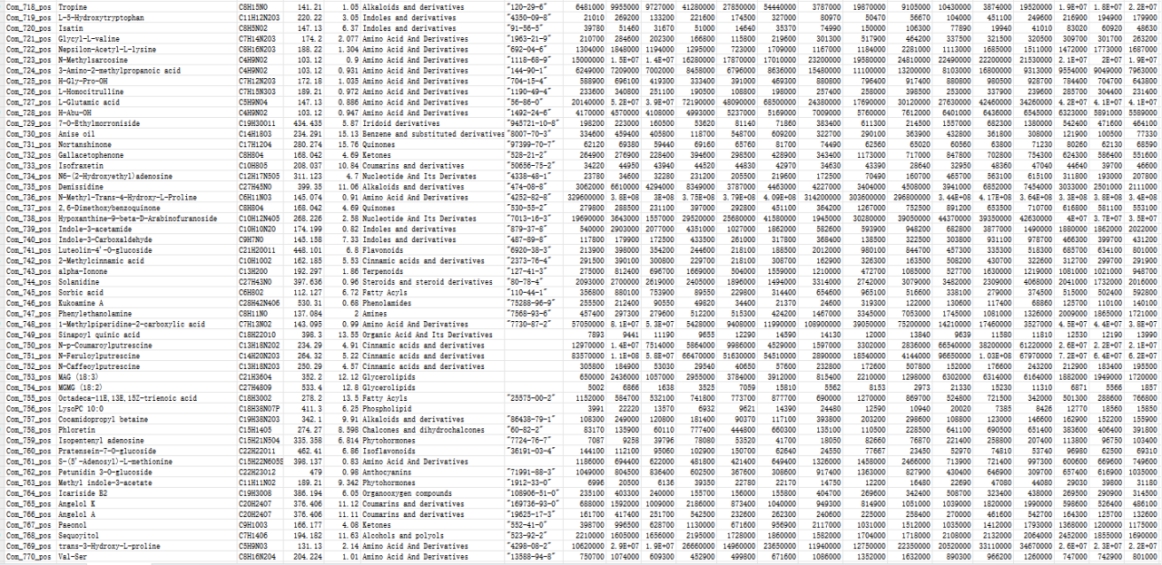

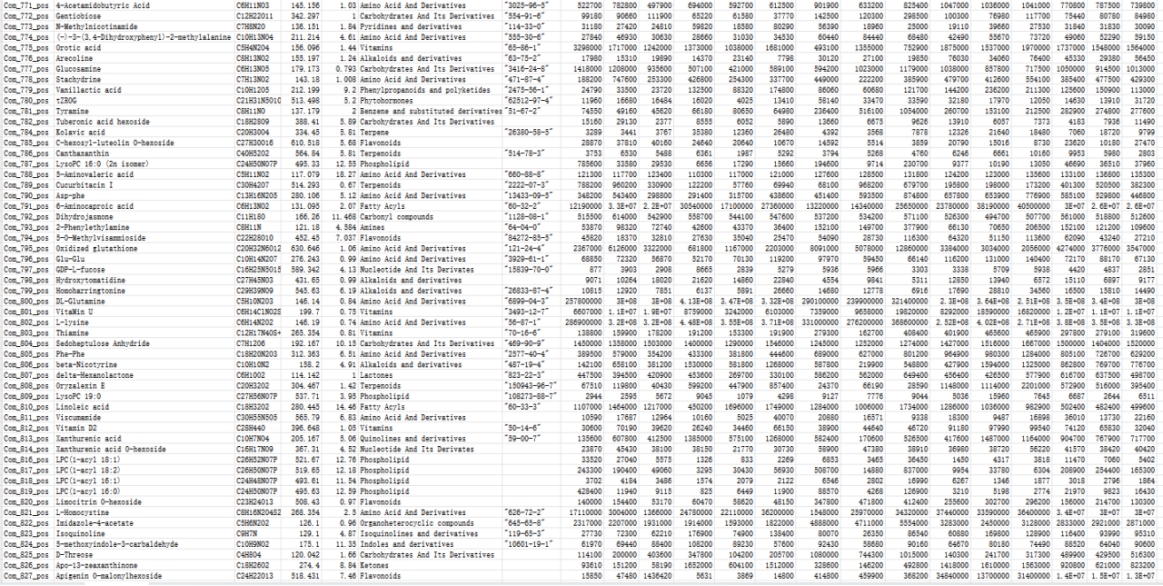

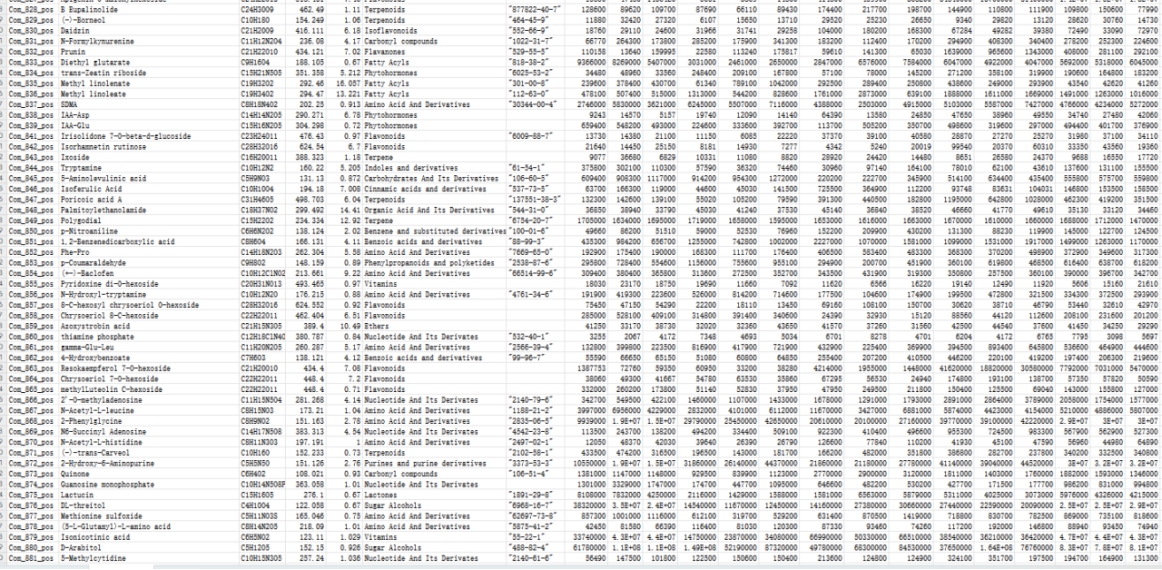

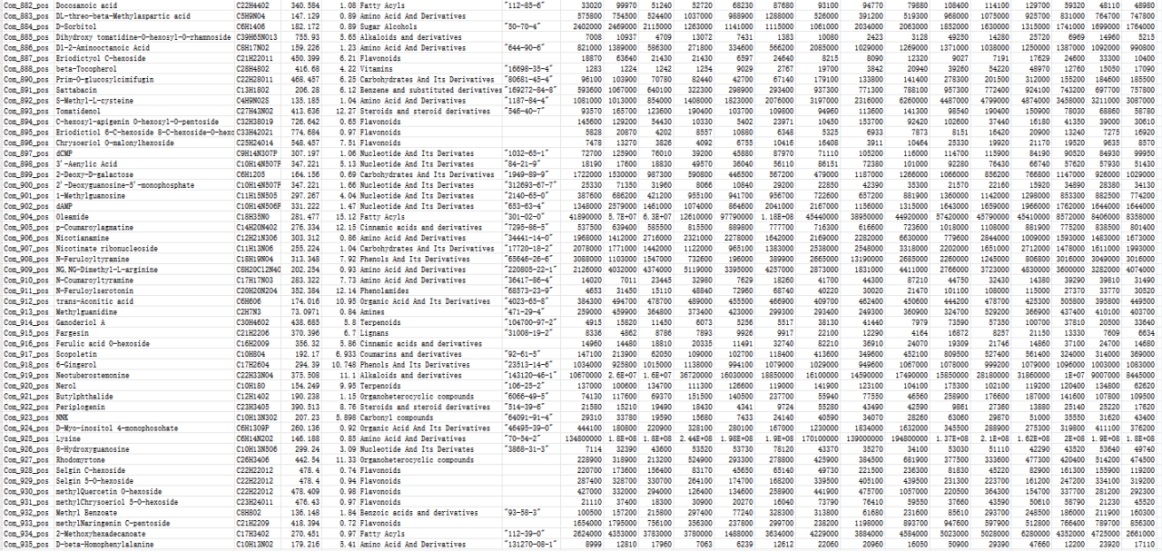

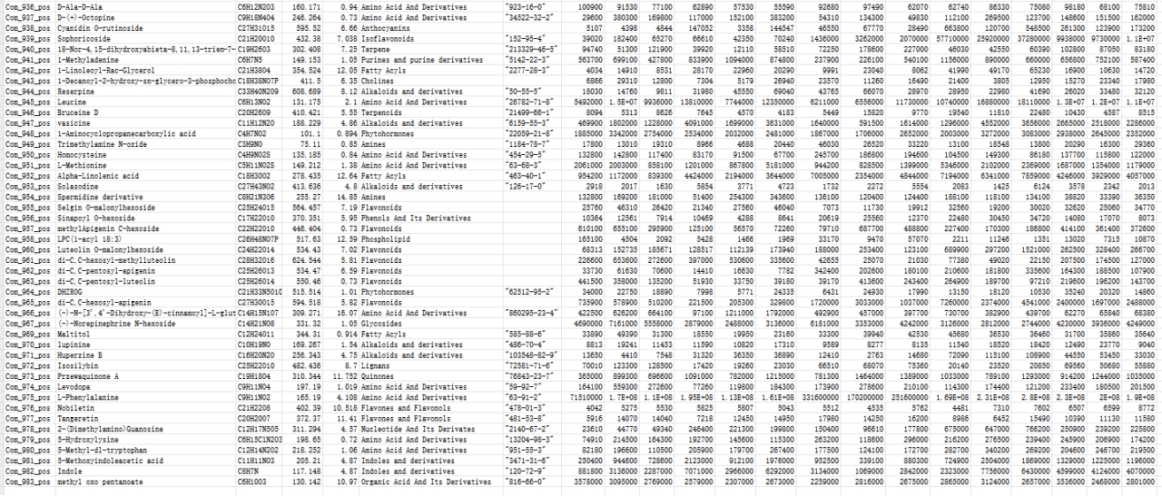

Supplement: Supplementary file 1 — Table S1. [file FSN3-13-e70571-s001.docx]
